# Characterization of the Specificity, Functionality, and Durability of Host T‐Cell Responses Against the Full‐Length Hepatitis E Virus

**DOI:** 10.1002/hep.28819

**Published:** 2016-10-28

**Authors:** Anthony Brown, John S. Halliday, Leo Swadling, Richie G. Madden, Richard Bendall, Jeremy G. Hunter, James Maggs, Peter Simmonds, Donald B. Smith, Louisa Vine, Cara McLaughlin, Jane Collier, David Bonsall, Katie Jeffery, Susanna Dunachie, Paul Klenerman, Jacques Izopet, Nassim Kamar, Harry R. Dalton, Eleanor Barnes

**Affiliations:** ^1^ Peter Medawar Building for Pathogen ResearchUniversity of OxfordOxfordUnited Kingdom; ^2^ The Royal Melbourne HospitalMelbourneVictoriaAustralia; ^3^ The Royal Cornwall HospitalTruroUnited Kingdom; ^4^ Oxford University Hospitals NHS Foundation TrustOxfordUnited Kingdom; ^5^ Centre for Immunity, Infection and Evolution, University of EdinburghUnited Kingdom; ^6^ Centre for Tropical Medicine & Global HealthUniversity of OxfordOxfordUnited Kingdom; ^7^ National Institute for Health Research (NIHR)Oxford Biomedical Research CentreOxfordUnited Kingdom; ^8^ The Rangueil HospitalToulouseFrance

## Abstract

The interplay between host antiviral immunity and immunopathology during hepatitis E virus (HEV) infection determines important clinical outcomes. We characterized the specificity, functionality, and durability of host T‐cell responses against the full‐length HEV virus and assessed a novel “Quantiferon” assay for the rapid diagnosis of HEV infection. Eighty‐nine volunteers were recruited from Oxford, Truro (UK), and Toulouse (France), including 44 immune‐competent patients with acute HEV infection, 18 HEV‐exposed immunosuppressed organ‐transplant recipients (8 with chronic HEV), and 27 healthy volunteers. A genotype 3a peptide library (616 overlapping peptides spanning open reading frames [ORFs] 1‐3) was used in interferon‐gamma (IFN‐γ) T‐cell ELISpot assays. CD4^+^/CD8^+^ T‐cell subsets and polyfunctionality were defined using ICCS and SPICE analysis. Quantification of IFN‐γ used whole‐blood stimulation with recombinant HEV‐capsid protein in the QuantiFERON kit. HEV‐specific T‐cell responses were detected in 41/44 immune‐competent HEV exposed volunteers (median magnitude: 397 spot‐forming units/10^6^ peripheral blood mononuclear cells), most frequently targeting ORF2. High‐magnitude, polyfunctional CD4 and CD8^+^ T cells were detected during acute disease and maintained to 12 years, but these declined over time, with CD8^+^ responses becoming more monofunctional. Low‐level responses were detectable in immunosuppressed patients. Twenty‐three novel HEV CD4^+^ and CD8^+^ T‐cell targets were mapped predominantly to conserved genomic regions. QuantiFERON testing demonstrated an inverse correlation between IFN‐γ production and the time from clinical presentation, providing 100% specificity, and 71% sensitivity (area under the receiver operator characteristic curve of 0.86) for HEV exposure at 0.3 IU/mL. *Conclusion:* Robust HEV‐specific T‐cell responses generated during acute disease predominantly target ORF2, but decline in magnitude and polyfunctionality over time. Defining HEV T‐cell targets will be important for the investigation of HEV‐associated autoimmune disease. (Hepatology 2016;64:1934‐1950).

**Abbreviations**
AA: amino acid
anti‐HEV: HEV antibody
AUROC: area under the receiver operator characteristic curve
BFA: Brefeldin A
CMV: cytomegalovirus
Cy: cyanin
DMSO: dimethyl sulfoxide
ELISA: enzyme‐linked immunosorbent assay
HEV: hepatitis E virus
HLA: human leukocyte antigen
ICS: intracellular cytokine staining
IFN‐γ: interferon‐gamma
Ig: immunoglobulin
IL: interleukin
Mip‐1‐β: macrophage inflammatory protein 1 beta
NK: natural killer
ORF: open reading frame
PB: Pacific Blue
PBMCs: peripheral blood mononuclear cells
PCR: polymerase chain reaction
PE: phycoerythrin
PFA: paraformaldehyde
PO: Pacific Orange
RT‐PCR: reverse‐transcriptase PCR
SFU: spot‐forming units
Tcm: central memory T cells
Tem: effector memory T cells
TNF‐α: tumor necrosis factor alpha

Hepatitis E virus (HEV) infects approximately 20 million people, claims 70,000 lives annually,^1^ and is a major cause of acute hepatitis in developing countries. In recent years, HEV has also been recognized as endemic in industrialized countries where seroprevalence rates as high as 50% have been reported and sporadic cases are common.^2^, ^3^ Although acute HEV infection is usually self‐limiting, it may present as a severe acute hepatitis associated with significant morbidity.

HEV is an RNA virus with a 7.2‐kilobase‐long, single‐stranded positive sense genome for which seven genotypes have been described.^1^, ^2^, ^3^, ^4^, ^5^ The virus contains three open reading frames (ORFs 1‐3): ORF1 encodes a ∼1,700‐amino‐acid (AA) nonstructural polyprotein; ORF2 encodes the 660‐AA capsid protein, and ORF3, which overlaps ORF2, encodes a 123‐AA phosphoprotein that has pleiotropic regulatory effects on host cells.^6^, ^7^, ^8^, ^9^ HEV genotypes 1 and 2 are responsible for most cases of infection in developing countries and cause large‐scale, water‐borne epidemics of acute hepatitis. Genotypes 3 and 4 HEV are zoonoses carried predominantly by domestic swine and wild boar and are the main cause of sporadic infection in humans in developed countries and in Asian developing countries (reviewed in Khuroo^4^ and Khuroo et al.^10^).

Several clinical observations highlight the interplay between effective host immunity, immunopathology, and baseline hepatic functional reserve that determine the clinical outcomes of HEV infection. Mortality rates in HEV genotypes 1 and 2 are especially high if infection occurs during pregnancy (15%‐29%)^11^, ^12^; the reasons for this are not clear, but likely, in part, relate to an altered host immune state in response to HEV viraemia, with data suggesting a skewing toward a Th2 cytokine response.^13^ Mortality rates are also high in patients with preexisting chronic liver disease; this is assumed to relate to a limited hepatic reserve in the face of adaptive immune responses against HEV.^14^, ^15^, ^16^ A reduction in hepatic reserve may also explain, in part, why HEV in the West commonly presents in older males who are more susceptible to presentation with jaundice, in the presence of other liver diseases including alcohol‐associated liver disease and diabetes in association with HEV adaptive immune responses.^17^ However, the importance of the adaptive immune system in controlling HEV infection is most clearly highlighted by the recent observation that HEV infection may cause persistent infection in immunosuppressed patients,^18^, ^19^, ^20^, ^21^, ^22^, ^23^, ^24^, ^25^ with a reduction in immunosuppressive therapy leading to HEV clearance.^24^

In natural infection and after vaccination, HEV immunity does not necessarily confer life‐long protection given that secondary infection has been described.^26^, ^27^, ^28^ Although humoral immunity and B‐cell epitopes against HEV have been extensively examined,^29^, ^30^, ^31^, ^32^, ^33^, ^34^ very few studies have evaluated the role of host cellular immune responses during acute and chronic HEV infection.^24^, ^35^, ^36^, ^37^, ^38^ Understanding the role that cellular immunity plays in clearing primary HEV infection may explain the limited durability of immune responses against HEV. To date, T‐cell studies of HEV infection have generally used proliferation assays that assess T‐cell responses against limited regions of the corresponding HEV genome (ORF2 and ORF3 proteins), and the durability of T‐cell responses in HEV‐infected subjects is not known. As a consequence, the full breadth and functional profile of T‐cell responses against HEV infection has not been established. Furthermore, although multispecific T‐cell responses against both ORF2 and ORF3 proteins have been demonstrated, no individual T‐cell epitopes have, as yet, been identified. This may be important given the association of HEV infection with autoimmune hepatic and neurological diseases, which may act through molecular mimicry.^39^, ^40^

In the present study, we aimed to better characterize the specificity, breadth, functionality, and durability of host T‐cell responses against HEV infection and provide further evidence for the role of host cellular immunity in viral clearance. We generated a comprehensive peptide library corresponding to the entire HEV genotype 3 genome and evaluated this in patients with acute, resolved, and chronic HEV infection. By fine mapping of positive interferon‐gamma (IFN‐γ) ELISpot responses, we identified the individual peptide targets of the HEV T‐cell responses and constructed pair‐wise distance plots to measure HEV variability at sites corresponding to the immunogenic T‐cell targets. We used intracellular cytokine staining (ICS) to determine T‐cell subsets and functionality. Finally, given that the sensitivity of existing serological tests for the diagnosis of HEV has been questioned,^41^, ^42^ we evaluated the utility of a novel Quantiferon‐based assay for detection of HEV specific T‐cell immunity for the diagnosis HEV infection.

## Patients and Methods

### CHARACTERISTICS OF STUDY SUBJECTS

All subjects included in the study gave written informed consent and were recruited from one of four sites: The John Radcliffe Hospital, Oxford (Oxford Research Ethics Committee 04.OXA.010) Centre for Clinical Vaccinology and Tropical Medicine; Oxford (National Research Ethics Service [Berkshire]; reference: 13/SC/0023); The Royal Cornwall Hospital, Truro (South West Ethics Committee; ref. no.: IRB‐06Q2101/61); and The Rangueil Hospital, Toulouse (institutional review board of Toulouse). Peripheral blood mononuclear cells (PBMCs) and serum were collected prospectively for each subject. Ethical approval for the study was obtained from the local ethics committee at each site.

A total of 89 subjects were included (Table 1) consisting of 18 immunosuppressed organ transplant recipients (Supporting Table S1) and 71 immune‐competent volunteers (Supporting Table S2). The immune‐competent subjects comprised two groups: (1) “HEV‐exposed” subjects (HEV immunoglobulin [Ig] G positive, ± HEV IgM positive, n = 44 [16 Truro and 28 Oxford]; median age, 65 years; range, 21‐92) and (2) “HEV‐unexposed” subjects (HEV IgG negative, n = 27; median age, 66; range, 22‐74). All subjects in the HEV‐exposed group had presented with symptomatic HEV infection (either jaundice or general malaise) and abnormal liver biochemistry, with positive HEV IgM antibodies. No patient in the unexposed group had ever experienced an episode of unexplained jaundice or biochemical hepatitis to suggest the unlikely possibility of previous seronegative HEV infection.

**Table 1:** Subject Baseline Characteristics

	Immune‐Competent Volunteers		Immunosuppressed Patients	
	HEV Exposed (n = 44)	HEV Unexposed (n = 27)	Transplant Chronic HEV (n = 8)	Transplant Resolved HEV (n = 10)
Age, years				
Median	65	66	49	55
Range	21‐92	22‐74	32‐67	30‐77
Sex				
Male:female	30:14	15:12	8:0	8:2
Genotype 3 (%)	28/44 (64)	—	8/8 (100)	8/10 (80)
Genotype 1 (%)	1/44 (2)		N/A	N/A
Unknown (%)	16/44 (34)		N/A	2/10 (20)
Time from onset of HEV infection (months)				
Median	6	—	24	20
Range	0.3‐139	—	2‐90	5‐35

Exposed = HEV IgG^+^ by ELISA at the time of PBMC sampling.

N/A, not applicable.

All 18 patients in the immunosuppressed group were receiving immunosuppressive therapy following organ transplantation (15 renal and 3 liver) at the time of recruitment (Supporting Table S1). Ten of these patients had a history of resolved HEV infection (anti‐HEV IgG positive and HEV negative by polymerase chain reaction [PCR]) and 8 had chronic HEV infection at the time of sampling (median HEV viral load by PCR, 7.2 × 10^5^ copies/mL; range, 1.8 × 10^5^ to 8.5 × 10^6^ copies/mL). All subjects were negative for hepatitis C virus antibody and hepatitis B surface antigen. HEV serological testing was performed using the commercially available HEV enzyme‐linked immunosorbent assay (ELISA) kit (Wantai Technologies, Beijing, China), according to the manufacturer's instructions.

### HEV VIREMIA AND GENOTYPING

For the samples from volunteers with a history of acute HEV infection, HEV genotyping was performed at Public Health England Reference Laboratories or performed in‐house by nested reverse‐transcriptase PCR (RT‐PCR) on 10 μL of purified RNA using the Access kit (Promega, Southampton, UK) with primers as described.^43^ In 1 patient, HEV viremia was detected by RNA sequencing analysis as described.^44^ For the samples from chronically infected patients, HEV RNA was detected by a nested RT‐PCR as described.^45^ Nucleotide sequences were deduced from bulk PCR products and genotyped by comparison with HEV genotype 3 subtype reference sequences.^46^

### DESIGN OF HEV PEPTIDE LIBRARY

A set of 616 peptides, 15 AAs in length, overlapping by 11 AAs, was designed using a full‐length genotype 3a sequence as a template (accession: HQ389543; Mimotopes, Victoria, Australia).

Peptides were initially dissolved in dimethyl sulfoxide (DMSO) and arranged into 12 pools based on the subunits of the viral genome (A‐I, L‐N; range, 25‐74 peptides/pool; see Supporting Table S3). Pools were used at a final concentration of 3 μg/mL of each peptide within the pool for IFN‐γ ELISpot or 1 μg/mL (each single peptide) for ICS.

### IFN‐γ ELISpot ANALYSIS

*Ex vivo* IFN‐γ ELISpot assays using HEV peptide pools were performed, according to the manufacturer's instructions (Mabtech, Sweden), on either frozen or freshly isolated PBMCs plated in duplicate at 2 × 10^5^ per well and incubated at 37°C for 16‐18 hours. Background wells (medium only and PBMCs + DMSO) were typically zero to four spots. The final concentration of DMSO in negative control wells and peptide antigen wells was 0.7%. Internal positive controls included concanavalin A (Sigma‐Aldrich, Poole, UK), peptide array NR‐9308 (mixed human leukocyte antigen [HLA] class I‐restricted peptides from influenza, Epstein‐Barr virus, and cytomegalovirus [CMV]) (BEI Resources, VA), and a CMV lysate (Virusys Corp, MD). Total HEV response was calculated by summing responses to all positive pools after subtracting DMSO background response from each positive pool. To define CD4^+^ and CD8^+^ T‐cell subsets, IFN‐γ ELISpot assays were performed after CD8^+^ T‐cell depletion using a CD8^+^ Dynabeads (Invitrogen, CA) kit according to the manufacturer's instructions.

### MAPPING OF HEV‐SPECIFIC T‐CELL RESPONSES

HEV‐specific T‐cell responses to peptide pools were initially mapped to minipools consisting of 8‐10 peptides, and positive responses were further mapped to individual 15‐AA peptides. Where responses were observed to consecutive peptides, the overlapping sequence (9‐11 AAs in length) between these peptides was synthesized (Mimotopes, Australia), and tested by IFN‐γ ELISpot assays. DNA extraction and HLA typing were performed as described.^47^ Epitope prediction was also performed *in silica* using NetMHC (http://www.cbs.dtu.dk/services/NetMHC) through analysis of the 15‐AA peptides in association with patients' corresponding HLA alleles.

### ICS

Thawed PBMCs were stimulated with either peptide pools or individual peptides (1 μg/mL for each peptide). Positive and negative controls were incubated with phorbol 12‐myristate 13‐acetate/ionomycin (50 and 500 ng/mL) or DMSO (0.12% by volume per pool, maximum of 5 pools and 0.58%), respectively. CD107α‐PE (phycoerythrin)/cyanin (Cy) 5 was added with peptide at the beginning of the assay. After overnight stimulation (Brefeldin A [BFA] was added after 1 hour at 10 mg/mL), cells were washed, fixed (1% paraformaldehyde [PFA]), and permeabilized (eBiosciences permeabilization buffer) and stained for 30 minutes with the following antibodies: CD3‐PO (Pacific Orange); CD4/Qdot605; CD8/PB (Pacific Blue); IFN‐γ/Alexa Fluor 700; interleukin (IL)‐2/allophycocyanin; tumor necrosis factor alpha (TNF‐α)/PE/Cy7; macrophage inflammatory protein 1 beta (Mip‐1‐β)/PE; and fixable near infrared live/dead dye (Invitrogen).

For phenotypic analysis, thawed PBMCs were rested overnight at 37°C + CO_2_, washed twice in PBS, and stained for 30 minutes at room temperature with the following antihuman antibodies: CD127/Pe/Cy7; CD45RA/peridinin chlorophyll protein complex/Cy5.5; and C‐C chemokine receptor type 7/PE. PBMCs were then washed and stimulated in RH10 for 5 hours with HEV peptide pools (1 μg/mL for each peptide). Positive and negative controls were as above. After stimulation (BFA was added after 1 hour at 10 mg/mL), cells were washed, fixed (1% PFA), and permeabilized (eBiosciences permeabilization buffer) and stained with the following antibodies at room temperature for 30 minutes: CD3/PO; CD4/Qdot605; CD8/PB; IFN‐γ/Alexa Fluor 700; and fixable near infrared live/dead dye (Invitrogen).

Flow cytometry was performed using a BD LSRII and analysis by FlowJo (Tree Star). Analysis of polyfunctionality was performed using Pestle and SPICE (version 5.3), downloaded from http://exon.niaid.nih.gov. All ICS data are corrected for background. The limit of detection by ICS equates to an IFN‐γ ELISpot response of ∼150 spot‐forming units (SFU)/10^6^ PBMCs; therefore, to allow HEV‐specific T‐cell response to be detectable, pools were combined if individual pool responses by ELISpot were less than 150 SFU/10^6^ PBMCs.

### GENERATION OF PHYLOGENETIC TREE AND PAIR‐WISE DISTANCE ANALYSIS

HEV complete genome sequences were downloaded from GenBank and aligned using SSE (v1.2).^48^ Human‐derived HEV genotype 3 isolates that differed from one another at >2% of nucleotide positions were assigned to a subtype and used to produce a neighbor joining tree using MEGA6.^49^ The distribution of pair‐wise AA distances among these sequences was computed using SSE.

### QuantiFERON ASSAY

An HEV recombinant capsid protein, HEV‐239, was generated under good manufacturing practices conditions, as described (purity, >95%),^50^ and used to stimulate whole blood in an overnight incubation. The IFN‐γ produced in response to stimulation was assessed by ELISA using the commercially available QuantiFERON kit, according to the manufacturer's instructions. HEV antigen 239 is a truncated capsid protein corresponding to AAs 368‐606 of the HEV ORF2 and is the key component of the current Innovax HEV vaccine.^51^ A null control was used for each sample as a negative control and subtracted from the HEV‐239 stimulated response, and a mitogen used as a positive control.

### STATISTICAL ANALYSES

Nonparametric tests were used, throughout, paired for within‐individual comparisons (Wilcoxon) and unpaired for group comparisons (Mann‐Whitney). Prism (v6.0 for Mac) was used throughout. A Pearson partial correlation was run using IBM SPSS (version 22) to determine the relationship between the total magnitude of the HEV‐specific T‐cell response by IFN‐γ ELISpot assay and the interval between the time of this assay and diagnosis, while controlling for age, sex, and viremic status. Data with a non‐Gaussian distribution (ELISpot response in SFU/10^6^ PBMCs and time of blood draw in months) were transformed to the logarithm of base 10.

## Results

### HEV GENOTYPING

In the immune‐competent HEV‐exposed group, 29 of 44 individuals tested positive for HEV RNA (Table 1). Of these, 28 were positive for HEV genotype 3 whereas 1 volunteer (029) was positive for genotype 1. In the immunosuppressed group, 16 of 18 patients tested positive for genotype 3 HEV RNA (subtype as shown; Supporting Table S1).

### ASSESSMENT OF HEV‐SPECIFIC T‐CELL RESPONSES BY IFN‐γ ELISpot ASSAY IN UNEXPOSED (HEV‐IgG‐NEGATIVE) IMMUNE‐COMPETENT VOLUNTEERS

**Figure 1:**
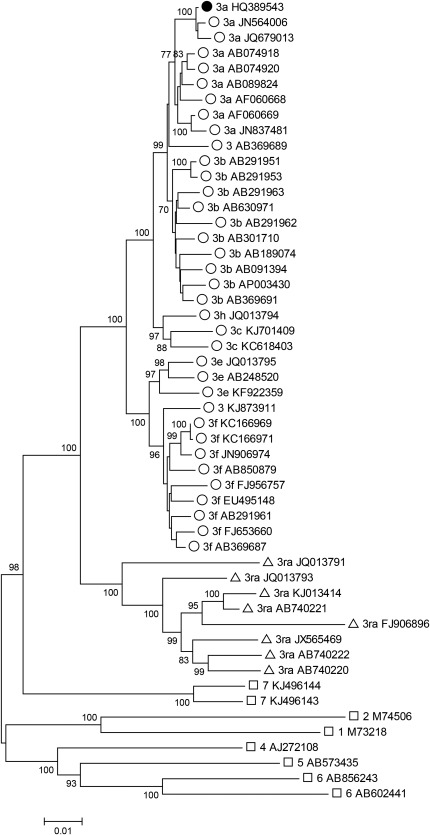
Phylogenetic analysis of HEV complete genome sequences. A neighbor‐joining tree was produced using a Poisson model of AA distances between 36 concatenated ORF1 and ORF2 regions (but not including the ORF1 hypervariable region) from human‐derived HEV genotype 3 isolates (open circles), rabbit‐derived genotype 3 isolates (open triangles), and reference sequences of other genotypes (open squares). Sequence HQ389543 (subtype 3a) that was used to generate the peptide set is shown with a filled circle. Branches supported by >70% of bootstrap replicates are indicated.

The only full‐length genotype 3 HEV sequence obtained from a patient in the UK was used to design the peptide panel. Although genotype 3 is diverse with 10 named subtypes and nine distinct, but unnamed, variants,^46^ phylogenetic analysis using all published HEV sequences showed that this sequence was a representative genotype 3a sequence (Fig. 1). To determine the specificity of our HEV peptide library, we first assessed T‐cell responses in 27 unexposed (HEV‐IgG‐negative), immune‐competent individuals by IFN‐γ ELISpot assay. Among these volunteers, T‐cell responses were very low, with a mean value of 6.3 SFU/10^6^ PBMCs per pool (Supporting Fig. S1A,B). One volunteer had a strong IFN‐γ ELISpot response to pool E (178 SFU/10^6^ PBMCs) that mapped to an individual peptide (HQRFPEAFYWTEFIM); no other unexposed or exposed individual responded to this epitope. The mean background response to DMSO was 9.4 SFU/10^6^ PBMCs.

### ASSESSMENT OF HEV‐SPECIFIC T‐CELL RESPONSES BY IFN‐γ ELISpot ASSAY IN HEV‐EXPOSED (HEV‐IgG‐POSITIVE) IMMUNE‐COMPETENT SUBJECTS

**Figure 2:**
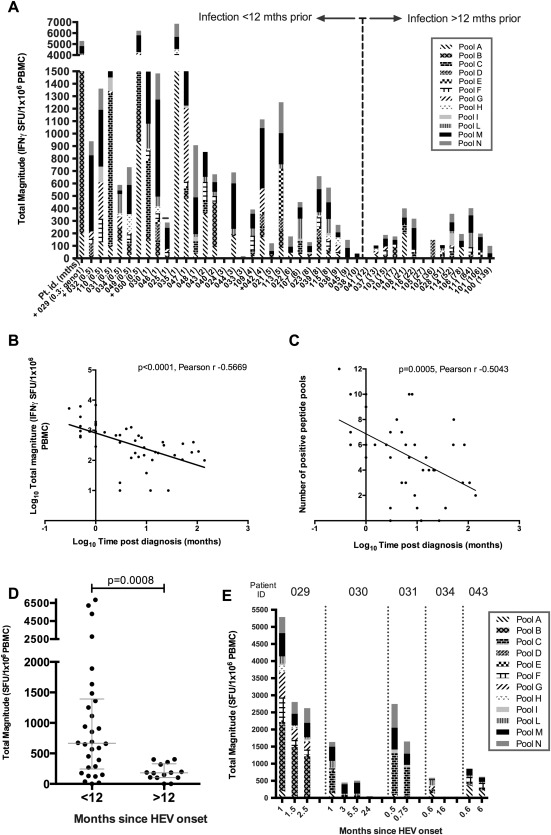
Total magnitude of HEV specific T‐cell responses in immune‐competent HEV‐exposed subjects measured by IFN‐γ ELISpot assay. (A) IFN‐ γ ELISpot responses displayed according to time interval (<12 months or >12 months) between diagnosis and assessment of T‐cell responses. x‐axis: individual subject number; the number in brackets represents the time interval (in months). Individuals who had detectable virus (PCR) at the time of the assay are indicated with a + on the x‐axis label, and the 1 patient infected with genotype 1 HEV (029) is labeled “geno1.” y‐axis: Each column represents the summed IFN‐γ ELISpot response to HEV peptide pools (A‐I, L‐N) for an individual patient. Individual shading patterns within bars indicate the contributing response of each peptide pool (see legend). (B) Pearson's correlation analysis of total magnitude of IFN‐γ ELISpot response (y‐axis; log_10_ scale) with increasing time from diagnosis in months (x‐axis; log_10_ scale). Linear regression line has been plotted. (C) Pearson's correlation analysis for number of positive peptide pools (y‐axis; linear scale) with increasing time from diagnosis in months (x‐axis; log_10_ scale). Linear regression line has been plotted. (D) Summed IFN‐γ ELISpot responses in individuals grouped according to an interval of either <12 months or >12 months between HEV infection and assessment. Horizontal lines represent group medians (±interquartile range). (E) Longitudinal IFN‐γ ELISpot responses, plotted as total magnitude (y‐axis) assessed over time (indicated on x‐axis) in 5 individual volunteers after onset of acute HEV infection.

We next assessed IFN‐γ ELISpot responses in 44 immune‐competent HEV exposed subjects (HEV IgG positive; Fig. 2A). Forty‐one had detectable HEV‐specific T‐cell responses. The median number of positive pools (any response greater than background) per patient was 6 (range, 0‐12 pools), and the median summed IFN‐γ ELISpot response was 397 SFU/10^6^ PBMCs (range, 0‐6,854). Pool M (in ORF2) was most frequently targeted, whereas responses to pool D (ORF1) were rarely detected (39 of 44 and 7 of 44 patients, respectively).

A negative association between the time post‐HEV presentation and the total magnitude of the HEV‐specific T‐cell response was demonstrated by Pearson's correlation (Fig. 2B). A Pearson partial correlation analysis was then performed to assess the impact of time postdiagnosis, presence of HEV RNA at time of analysis, age, and sex on the magnitude of the HEV‐specific T‐cell response. There was a moderate, negative correlation between the magnitude of the HEV‐specific T‐cell response and the time postdiagnosis in our cohort while controlling for age, sex, and viremic status, which was statistically significant (r = −0.383; N = 44; *P* = 0.013). Although there was a statistically significant correlation between the magnitude of the HEV‐specific T‐cell response and viremic status (r = 0.338; N = 44; *P* = 0.025), this was no longer significant when controlling for time post‐HEV diagnosis. The breadth of the HEV‐specific T‐cell response, as measured by the number of positive HEV peptide pools, also showed a significant negative correlation with time post‐HEV diagnosis (Fig. 2C).

IFN‐γ ELISpot responses were significantly higher in immune‐competent exposed individuals who had a history of acute HEV infection within 12 months of blood sampling, compared with those greater than 12 months (median, 667 vs. 181 SFU × 10^6^ cells; Mann‐Whitney U test, *P* = 0.0008; Fig. 2D). Longitudinal IFN‐γ ELISpot responses were assessed in 5 patients who presented with very recent onset of symptomatic HEV infection, and this analysis showed a rapid decline in HEV‐specific T‐cell responses after the first weeks of follow‐up (Fig. 2E).

### IMMUNOSUPPRESSED PATIENTS WITH RESOLVED OR CHRONIC HEV INFECTION

**Figure 3:**
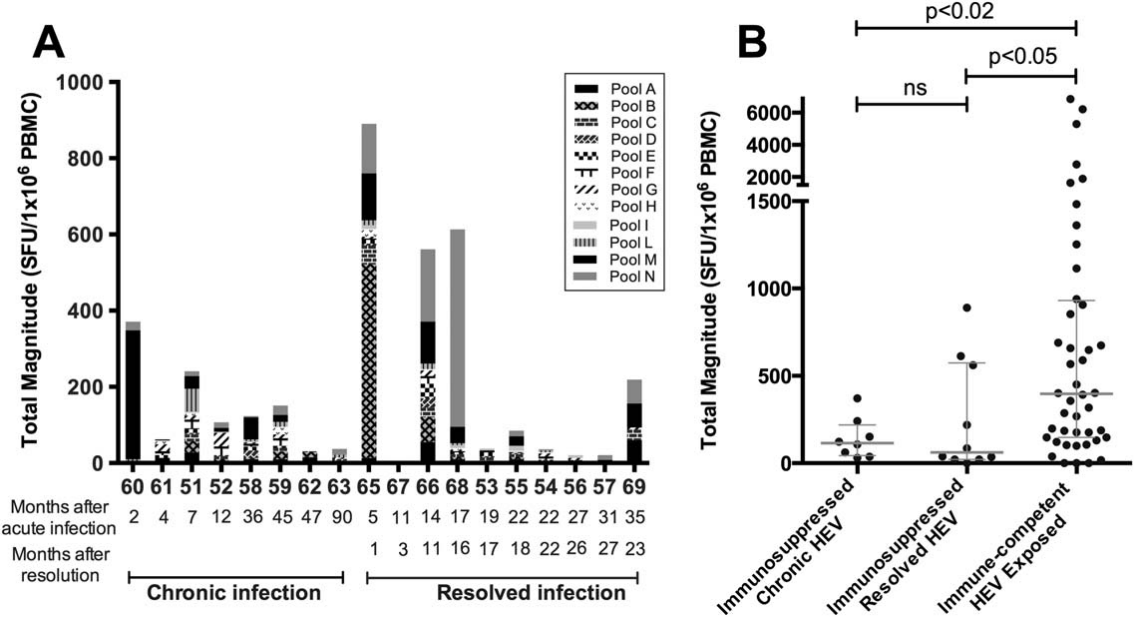
IFN‐γ ELISpot responses in immunosuppressed patients with resolved or chronic HEV infection. (A) Each bar represents the summed IFN‐γ ELISpot response for an individual patient to HEV peptide pools. Individual shading patterns within bars indicate the contributing response of each pool (see legend). x‐axis: individual patient numbers with the interval (in months) between diagnosis of acute infection and sampling shown below. In patients who resolved HEV infection, the number of months since clearance at time of sampling is also shown. (B) Immunosuppressed patients with either chronic HEV infection (HEV^+^ by PCR) or resolved HEV infection (HEV IgG^+^, HEV^‐^ by PCR) compared with total HEV response in 44 immune‐competent HEV‐exposed individuals. Horizontal lines represent group medians (±interquartile range). Abbreviation: ns, not significant.

To characterize cellular immune responses against HEV infection in immunosuppressed patients, we next assessed IFN‐γ ELISpot responses in 18 organ transplant recipients receiving immunosuppressive therapy (Supporting Table S1). Of these, 8 patients had evidence of chronic HEV infection (HEV positive by PCR) at the time of assessment, whereas the remaining 10 were infected posttransplantation and had resolved HEV infection at the time of sampling (Fig. 3). Compared with responses in HEV‐exposed immune‐competent individuals, responses in immunosuppressed patients were significantly lower (397 vs. 96 SFU × 10^6^ PBMCs; median values, *P* < 0.01). We did not observe a statistically significant difference between the magnitude of responses in immunosuppressed patients with chronic infection compared with those who had previously resolved infection (*P* = 0.74, Mann‐Whitney; Fig. 3B).

### FINE MAPPING OF ANTI‐HEV T‐CELL RESPONSES

We identified the individual peptide targets of anti‐HEV T‐cell responses using IFN‐γ ELISpot assays. Fine mapping was performed by repeat testing of positive IFN‐γ ELISpot pools using smaller pools of peptides (minipools) and individual peptides contained within these. Using this approach, we identified 40 individual 15‐AA‐long peptides targeted by HEV‐specific T‐cell responses (Table 2A). Assuming that positive overlapping peptides contained a single T‐cell target, we identified 23 T‐cell targets in total, nine of which were further mapped to 11‐AA‐long peptides (Table 2A,B). Responses to 14 of the identified targets were detected in more than 1 HEV exposed subject. In addition, using knowledge of the responding patient(s) HLA type and the experimentally defined 15‐AA‐long peptide T‐cell targets, *in silico* epitope prediction was performed using program NetMHC and resulted in a further 12 T‐cell epitopes (Supporting Table S4).

**Table 2A:** HEV T‐Cell Target Mapping to Individual 15 Amino Acid Peptides

HEV Peptide No.	HEV Peptide Sequence	HEV Peptide Pool	HEV Genome Region	Responding Patient(s) (SFU/10^6^ PBMC)	CD4/CD8 Restriction
10	FLSRLQTEILINLMQ	A	ORF1	031 (25)	Un
38	GFSRCAFAAETGVAL	A	ORF1	035 (1370), 043 (295)	CD8
39	CAFAAETGVALYSLH			043 (135)	
78	KSTFHAVPVHIWDRL	B	ORF1	029 (1098)	CD8
79	HAVPVHIWDRLMLFG			029 (830)	
113	LVFDESVPCRCRTFL	C	ORF1	030 (50), 031 (1055)	CD8
114	ESVPCRCRTFLKKVA			030 (70), 031 (955)	
199	RRLLYTYPDGAKVYA	E	ORF1	113 (130)	CD8
200	YTYPDGAKVYAGSLF			113 (95)	
260	NGRRVVIDEAPSLPP	F	ORF1	029 (93), 035 (105)	CD4
281	TSRVLRSLFWNEPAI	F	ORF1	043 (118), 109 (80), 110 (98)	CD8
282	LRSLFWNEPAIGQKL			109 (63), 110 (75)	
328	CRMAAPSQRKAVLST	G	ORF1	029 (205), 034 (75), 042 (30), 047 (25), 029 (198), 110 (100)	Un
329	APSQRKAVLSTLVGR				
330	RKAVLSTLVGRYGRR				
432	CFCLCCPRHRPASRL	I	ORF3	031 (45)	Un
433	CCPRHRPASRLAVVV			031 (35)	
456	LFFVFLPMLPAPPAG	L	ORF2	030 (15), 034 (40)	Un
466	QPFALPYIHPTNPFA	L	ORF2	029 (18), 043 (33), 109 (68), 115 (30), 029 (15)	Un
467	LPYIHPTNPFAADVV				
486	GAILRRQYNLSTSPL	M	ORF2	029 (35), 032 (20), 045 (30), 110 (55) 115 (50), 029 (70), 030 (15), 035 (115), 045 (45), 109 (33), 110(50), 029 (40), 030 (45)	CD4
487	RRQYNLSTSPLTSSV				
488	NLSTSPLTSSVASGT				
491	SGTNLVLYAAPLNPL	M	ORF2	031 (80), 032 (85)	CD8
492	LVLYAAPLNPLLPLQ			031 (30), 035 (210)	
514	ASELVIPSERLHYRN	M	ORF2	035 (90), 108 (30), 110 (120)	CD4
521	EEATSGLVMLCIHGS	M	ORF2	101 (723)	CD8
525	VNSYTNTPYTGALGL	M	ORF2	030 (110), 031 (523), 045 (60), 047 (340), 101 (53), 113 (55), 030 (90), 031 (525), 045 (30), 101 (65)	CD8
526	TNTPYTGALGLLDFA				
539	LTTTAATRFMKDLHF	M	ORF2	042 (135)	CD8
550	TELISSAGGQLFYSR	N	ORF2	035 (290), 108 (40)	CD8
555	NGEPTVKLYTSVENA	N	ORF2	029 (50)	CD4
556	TVKLYTSVENAQQDK			029 (135)	
567	TPSPAPSRPFSVLRA	N	ORF2	031 (110), 045 (35)	CD8
576	TNPMYVSDTVTFVNV	N	ORF2	031 (35)	CD8
577	YVSDTVTFVNVATGA			031 (25)	
587	SKTFYVLPLRGKLSF	N	ORF2	031 (120)	CD4
588	YVLPLRGKLSFWEAG			031 (40)	
603	AVGVLAPHSALAVLE	N	ORF2	031 (115)	CD8
604	LAPHSALAVLEDTVD			031 (105)	

Regions of sequence overlap between adjacent peptides are shown underlined.

CD4 or CD8 restrictions were determined by CD8^+^ depletion ELISpot.

Abbreviation: Un, unknown.

**Table 2B:** HEV Epitope Mapping to 11 Amino Acid Peptides by ELISpot Assay

HEV Peptide No.	HEV T‐Cell Epitope Sequence	HEV Peptide Pool	HEV Genome Region	Responding Patient(s) (SFU/10^6^ PBMC)
38/39	CAFAAETGVAL	A	ORF1	35 (1248)
				43 (298)
78/79	HAVPVHIWDRL	B	ORF1	29 (240)
113/114	ESVPCRCRTFL	C	ORF1	30 (25)
199/200	YTYPDGAKVYA	E	ORF1	113 (293)
281/282	LRSLFWNEPAI	F	ORF1	43 (153)
				110 (70)
432/433	CCPRHRPASRL	I	ORF3	31 (25)
466/467	LPYIHPTNPFA	L	ORF2	43 (30)
				115 (23)
525/526	TNTPYTGALGL	M	ORF2	30 (108)
				45 (58)
				113 (70)
576/577	YVSDTVTFVNV	N	ORF2	31

### HEV SEQUENCE DIVERSITY AT SITES CORRESPONDING TO PEPTIDE TARGETS OF ANTI‐HEV T‐CELL RESPONSES

**Figure 4:**
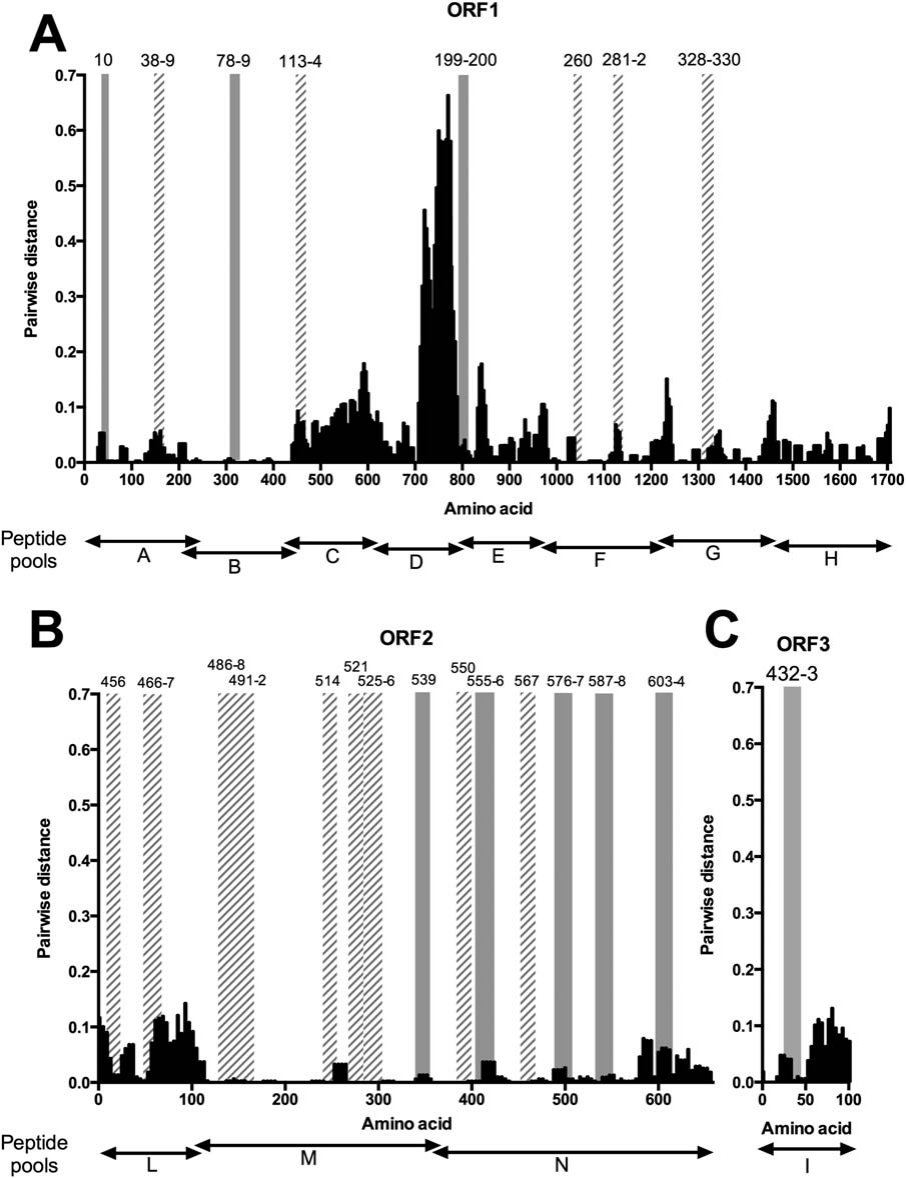
Location of reactive peptides and AA variability. The distribution of mean AA pair‐wise distances among concatenated human HEV‐3 ORF1, ORF2, and ORF3 sequences is shown for each 15‐mer peptide. The same human genotype 3 sequences were used as in Fig. 1. ORFs 1‐3 are displayed in separate plots A‐C respectively. Vertical bars indicate the position of reactive peptides detailed in Table 2A; peptides that were targeted by a single volunteer are shown by solid gray bars whereas those targeted by multiple volunteers have diagonally striped bars. The peptide pool boundaries (A‐I and L‐N) are shown below each graph. The x‐axis indicates amino acid position in each ORF relative to AF082843.

To establish the relative level of diversity for each ORF of the HEV genome at immunogenic peptide targets identified by IFN‐γ ELISpot mapping, we next generated pair‐wise distance plots (Fig. 4) using the 36 full‐length human HEV genotype 3 sequences that were used to generate Fig. 1. Most peptide responses were directed against regions of the genome that are relatively well conserved, with the majority located in ORF2, which encodes the main structural subunits of the virus. None of the epitopes that we identified targeted a region of hypervariability within ORF1 (AA numbers 707‐792, numbered relative to AF082843).

### CYTOKINE PROFILE OF ANTI‐HEV T‐CELL RESPONSES AS ASSESSED BY ICS

**Figure 5:**
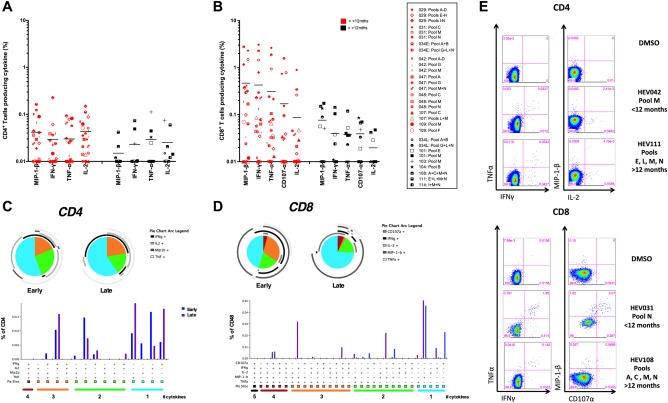
Functionality of T‐cell responses following HEV peptide stimulation (A,B); percentages of CD4^+^ (A) or CD8^+^ (B) T cells producing a given cytokine (background subtracted). Each symbol represents a patient's response to PBMC stimulation overnight (16 hours) with a peptide pool or pools assessed by intracellular cytokine staining. Fourteen immunocompetent HEV‐exposed individuals were assessed (for subject IDs, see legend). Symbols in red are subjects with an interval between diagnosis and collection of PBMCs of <12 months (n = 21); those in black are >12 months (n = 9). Horizontal lines represent group means. (C,D) Polyfunctionality of T‐cell responses to HEV peptide pool stimulation. SPICE analysis was performed on positive CD8^+^ and CD4^+^ T‐cell responses from 14 immunocompetent HEV‐exposed volunteers (responses >0.03% of CD4^+^ or CD8^+^ T cells producing any single cytokine assessed were considered positive) measured at an early time point (<12 months) or a late time point postdiagnosis (>12 months). Pie charts represent the proportion of cytokine‐secreting cells that produce one, two, three, and four cytokines (IFN‐γ, TNF‐α, MIP‐1‐β, and/or IL‐2) for CD4^+^ T cells and up to five cytokines (CD107α) for CD8^+^ T cells. Pie arcs show the proportion of T cells producing a single cytokine. Bar and pie base, median. (E) Example ICS FACS plots: staining for TNF‐α versus IFN‐γ and MIP‐1‐β versus IL‐2 for CD4^+^ T cells and TNF‐α versus IFN‐γ and MIP‐1‐β versus CD107α for CD8^+^ T cells after stimulation with pooled HEV peptides or DMSO (negative control). Plots are gated on live, CD3^+^, CD4^+^, or CD8^+^ T cells.

We determined the cytokine profile of HEV‐specific T‐cell responses by ICS following peptide stimulation with HEV peptide pools and individual immunogenic peptides identified by ELISpot mapping in 14 immune‐competent HEV exposed subjects. Both CD8^+^ and CD4^+^ responses were observed (Fig. 5). HEV‐specific CD8^+^ T‐cell responses were stronger and more readily detectable by ICS than CD4^+^ T‐cell responses both early (<12 months) and late (>12 months) after HEV presentation. However, both CD4^+^ and CD8^+^ T‐cell responses decreased in magnitude late after infection (Fig. 5A,B).

The polyfunctionality of HEV‐specific CD4^+^ T cells did not differ when measured early or late after diagnosis; however, a contraction of the IL‐2 monoproducing T cells and expansion of IFN‐γ monoproducing T cells was observed. A significant proportion of CD4^+^ T cells made IFN‐γ, TNF‐α, and IL‐2 at both time points (Fig. 5C). Early postinfection, approximately half of the HEV‐specific CD8^+^ T cells were MIP‐1‐β or IFN‐γ monoproducers, and half were polyfunctional—producing a combination of MIP‐1‐β and IFN‐γ, or MIP‐1‐β, IFN‐γ, and TNFα, with little IL‐2 or CD107α when stimulated with HEV peptides (Fig. 5B). In contrast, at later time points, the majority of CD8^+^ T cells were MIP‐1‐β monoproducers (Fig. 5D).

The memory phenotype (CD127, CD45RA, and CCR7) of HEV‐specific T cells was assessed early (1 month) and late (16‐22 months) after HEV infection. Both early and late postinfection, HEV‐specific T cells were predominantly CD127^+^ (1 month, median CD4^+^ 75.2% and CD8^+^ 46.15%; 16‐22 months, CD4^+^ 83.3% and CD8^+^ 51.1%; n = 2‐3; Supporting Fig. S2). HEV‐specific CD4^+^ and CD8^+^ T cells were central memory (Tcm) and effector memory (Tem) phenotype, with a shift from predominantly Tem to Tcm in CD4^+^ and an increase in Tem and Temra CD8^+^ T cells when comparing early to late time points post‐HEV infection (Supporting Fig. S2).

The change in phenotype and polyfunctionality of HEV‐specific T cells are characteristic of those observed for T‐cell memory differentiation after a cleared acute viral infection.^52^, ^53^, ^54^, ^55^

### QUANTIFICATION OF IFN‐γ PRODUCTION BY WHOLE BLOOD FROM HEV‐EXPOSED, IMMUNE‐COMPETENT VOLUNTEERS AFTER HEV ANTIGEN STIMULATION

**Figure 6:**
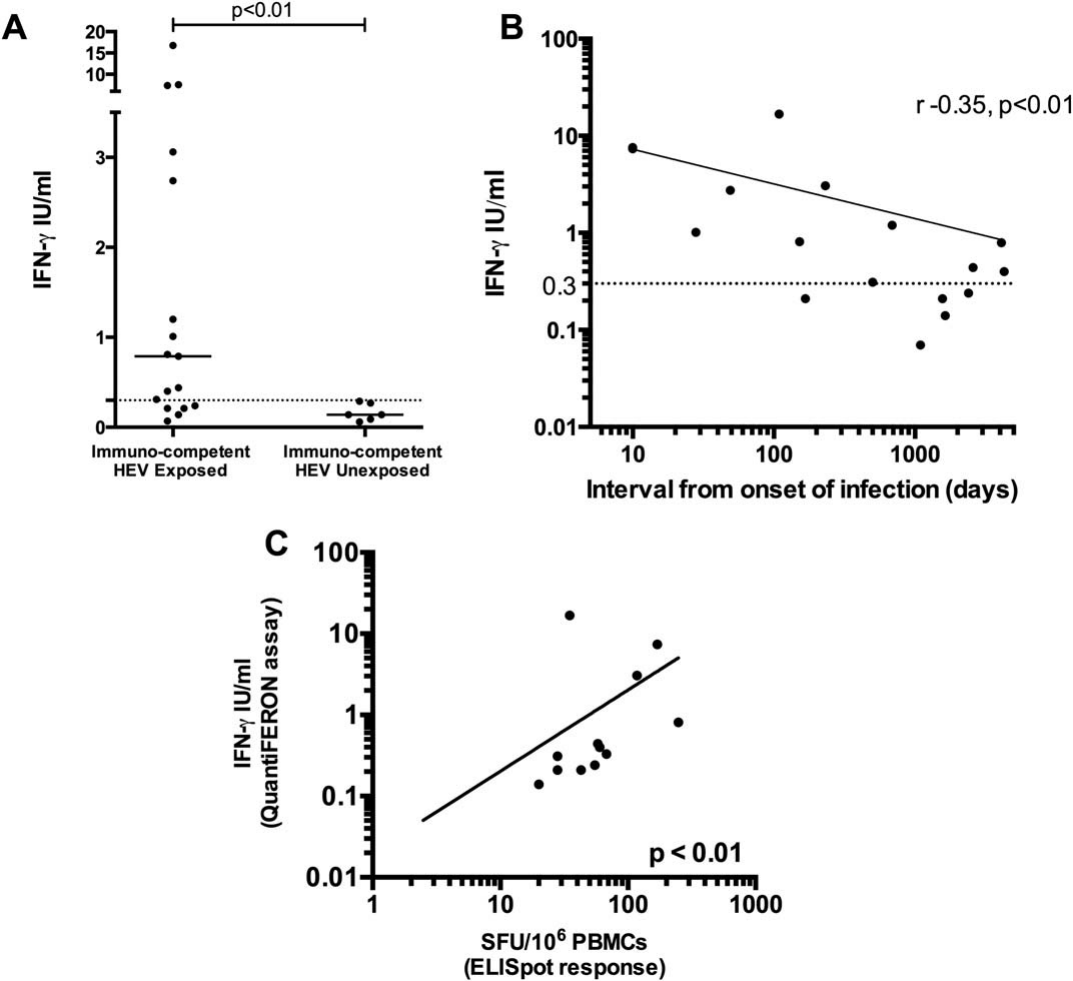
Quantitative assessment of IFN‐γ production by whole blood in response to HEV antigen 239. (A) Six unexposed healthy volunteers compared to 17 immune‐competent HEV‐exposed individuals. Complete horizontal lines represent group medians; the horizontal dotted line represents an arbitrary cut off of 0.3 IU/mL. (B) IFN‐γ production assessed using the QuantiFERON assay. The x‐axis (log scale) represents the time (days) between clinical onset of HEV infection and sample collection. The y‐axis (log scale) is the plasma IFN‐γ concentration after stimulation of whole blood *in vitro* with HEV antigen 239. (C) Spearman's correlation analysis between QuantiFERON results (y‐axis; log_10_ scale) and IFN‐γ ELISpot (x‐axis; log_10_ scale) response to the peptide pool (pool N) corresponding to HEV antigen 239. Lines of best fit are demonstrated on graphs (B) and (C).

Having shown that ORF2 was the most common T‐cell target after HEV exposure, we used a commercially available QuantiFERON assay kit to assess IFN‐γ production by whole‐blood samples after *ex vivo* stimulation with HEV antigen 239 in immune‐competent HEV‐exposed volunteers. HEV antigen 239 is a truncated capsid protein corresponding to AAs 368‐606 of the HEV ORF2 and is the key component of the current Innovax HEV vaccine.^51^ IFN‐γ production was significantly increased in HEV‐exposed volunteers compared with unexposed healthy volunteers after HEV 239 stimulation *ex vivo* (0.79 vs. 0.14 IU/mL; median values, *P* < 0.01; Fig. 6A). We also observed an inverse correlation between IFN‐γ production and the time from onset of clinical HEV infection (*P* < 0.001; Spearman r = ‐0.35; Fig. 6B). An arbitrary cut off of 0.3 IU/mL for this assay provides 100% specificity for HEV exposure in our cohort, with 71% sensitivity, and an area under the receiver operator characteristic curve (AUROC) of 0.86. Of the 17 volunteers who had HEV 239 antigen QuantiFERON assessment, 13 had simultaneous collection of PBMCs for comparison of IFN‐γ ELISpot responses (Fig. 6C). We observed a direct correlation between QuantiFERON‐measured IFN‐γ after HEV antigen 239 stimulation and IFN‐γ ELISpot responses to pool N (the pool containing the corresponding structural peptides; *P* < 0.01; Spearman r = 0.72).

## Discussion

In this study, we report an assessment of HEV‐specific T‐cell immunity against peptides encoded by the full‐length HEV genotype 3 genome, in both immune‐competent and immunosuppressed individuals with a history of HEV exposure. In immune‐competent HEV‐exposed individuals, we observed broad T‐cell responses targeting antigens from all three ORFs of the HEV genome. The majority of these had detectable T‐cell responses targeting multiple parts of the HEV genome. ORF2—the key component of two recently developed recombinant protein HEV vaccines^56^, ^57^ that codes for the viral capsid protein—was the most commonly targeted viral domain.

The durability of host HEV immunity after acute infection is unknown. Our data suggest that although T‐cell responses can be detected more than 10 years after primary HEV infection, the magnitude of these responses decreases in the early months after primary infection. This is largely based on cross‐sectional data; however, longitudinal analysis of T‐cell responses in 5 immune‐competent patients with acute HEV infection provides further evidence of the rapid contraction of anti‐HEV T‐cell responses with time. Others have shown that humoral responses against HEV can be detected more than 12 years after primary infection and that these contract over time (negative seroconversion rates 1.4%/year).^58^, ^59^ The capacity for secondary reinfection with HEV in some individuals^27^ may simply be a result of waning adaptive immune responses in the absence of HEV infection with a decline in CD8^+^ T‐cell polyfunctionality. However, other possibilities, such as infection with an alternative HEV genotype in the absence of cross‐reactive T‐cells, should also be considered.

Next, we identified 23 individual (17 overlapping) HEV‐specific T‐cell peptide targets in immune‐competent subjects, 14 of which were targets in more than 1 patient. We experimentally mapped nine of the CD8^+^ T‐cell targets to 11‐AA‐long peptides and used an *in silico* approach to predict 12 additional epitopes in association with host HLA alleles. We assessed the relative variability of the HEV genome at sites corresponding to immunogenic targets and showed that these were generally located in regions of the genome with low variability, with none located in the hypervariable region of ORF1. It is likely that these T‐cell targets would be cross‐reactive with other HEV genotypes, but this would require formal evaluation in future studies. Some evidence for intergenotypic T‐cell cross‐reactivity was provided by the high magnitude and broad T‐cell response observed in the HEV genotype 1‐infected patient (patient 029) assessed using genotype 3 peptides.

An association between acute HEV infection and autoimmune diseases, including Guillain‐Barre syndrome,^60^ neuralgic amyotrophy,^61^ and acute transverse myelitis,^62^ has been demonstrated. These associations are thought to be caused by molecular mimicry, whereby as a consequence of shared molecular homology, host immune responses against virus inadvertently target self‐antigen. This study now provides a series of HEV T‐cell targets that can be assessed in other diseases where HEV has been implicated in pathogenesis.

Assessed by ICS, we found that anti‐HEV memory T‐cell responses in immune‐competent HEV‐exposed subjects were predominantly CD8^+^ and monofunctional. CD8^+^ T cells most frequently produced MIP‐1‐β, a chemokine important for the recruitment of innate immune cells, followed by IFN‐γ, TNF‐α, and CD107‐α. CD4^+^ T cells, on the other hand, were more frequently positive for IFN‐γ, TNF‐α, and IL‐2. IL‐7 signaling is an important survival signal for memory T cells, and the expression of its receptor, CD127, is characteristic of long‐lived memory T cells.^52^, ^53^, ^54^, ^55^ HEV‐specific T cells assessed here were predominantly CD127^+^ and were a mixed population of central (Tcm) and effector memory (Tem) subsets, with little CD45RA reexpressing T cells. The phenotype and cytokine profile of the HEV‐specific T cells is indicative of a T‐cell response to an acute, cleared, viral infection, and it is consistent with the development of a population of memory T cells that may persist and offer enhanced protection on reinfection.

Compared with immune‐competent HEV‐exposed subjects, we found that organ transplant recipients had anti‐HEV T‐cell responses that were reduced in magnitude and breadth, irrespective of whether they had chronic or resolved HEV infection. In conflict with previously published data,^24^ we did not observe that immunosuppressed patients who had resolved their infection had significantly higher magnitude responses than those with chronic infection. One possible explanation for this discrepancy between studies may relate to the timing of T‐cell assessment relative to that of the resolution of HEV infection—assuming the same contraction of T‐cell responses observed in immune‐competent HEV‐exposed subjects occurs in immunosuppressed patients, those who had cleared infection would have reduced responses if assessed at significantly later time points. In our study, the median time between HEV onset and assessment was 24 months for organ transplant recipients, but data relating to timing of resolution of viremia were not available for all subjects.

We next assessed the diagnostic utility of a commercially available QuantiFERON assay that uses HEV antigen 239 (a truncated capsid protein) to confirm HEV exposure in immune‐competent subjects. Although there has been significant improvement in the diagnostic sensitivity of serological assays for HEV infection, the possibility that they lack specificity has been raised by reports of high HEV IgG seroprevalences in regions where acute HEV is diagnosed only rarely.^3^ In our study, all “exposed” HEV subjects had clinical evidence of HEV infection, although the possibility of seronegative, unrecognized infection in our healthy control group cannot be excluded. We observed increased IFN‐γ in HEV‐exposed, compared to unexposed, individuals and an inverse correlation between IFN‐γ production and time from onset of clinical HEV infection. Although an arbitrary cut off of 0.3 IU/mL for this assay provided 100% specificity for HEV exposure, this was only 71% sensitive, with an AUROC of only 0.86. Clearly therefore, this test has limited applicability for the confirmation of HEV exposure.

Finally, whereas our study was specifically designed to assess the role of HEV‐specific T cells in HEV control, viral peptides have also been shown to regulate natural killer (NK) activity.^63^ Whereas we have largely confirmed that the IFN‐γ responses we detect in our assays are T‐cell derived, we believe that the role of NK cells in controlling HEV should be evaluated in further studies.

In summary, we have demonstrated that the cellular immune response against HEV genotype 3 infection is broadly targeted against all three ORFs of the HEV genome in both immune‐competent and, to a more limited extent, immunocompromised subjects. Where measurable, HEV‐specific T cells target relatively conserved HEV peptides and are predominantly focused toward the HEV capsid protein. We demonstrate that T‐cell responses are still detectable more than 10 years after the resolution of HEV infection, and although these responses contract with time, their focus toward conserved viral antigens is likely to assist both in the clearance of primary HEV infection and, also, in the protective immune response against recurrent disease.

Author names in bold designate shared co‐first authorship.

## Supporting information

Additional Supporting Information may be found at onlinelibrary.wiley.com/doi/10.1002/hep.28819/suppinfo.

**Supplementary Figure 1:** Additional IFNg ELISpot data in HEV Exposed and Unexposed individuals **(A)** IFN‐ γ ELISpot response to individual peptide pools (A‐I, L‐N), DMSO (negative control) and Concanavlin A (positive control) in immune‐competent, HEV exposed (black dots) and HEV unexposed (open circles) subjects. Each point represents an individuals' response to the corresponding anitgen. Horizontal lines depict median responses. All points represent raw data, without background subtraction. (**B**) IFN‐ γ ELISpot response to individual peptide pools (A‐I, L‐N) in HEV unexposed subjects. DMSO (negative control) background response has been subtracted for each data point. Horizontal lines represent group medians (+/‐ IQR). (**C**) Pearson's correlation analysis for total magnitude of IFN‐γ ELISpot response (Y‐axis; log_10_ scale) with increasing age of individuals at time of sampling (X‐axis; linear scale). Semi‐log regression line has been plotted. (**D**) Pearson's correlation analysis for number of positive peptide pools (Y‐axis; linear scale) with increasing age of individuals at time of sampling (X‐axis; linear scale). Linear regression line has been plotted.
**Supplementary Figure 2: The memory phenotype of HEV‐specific T‐cells**: **A‐C**) Frozen PBMCs were thawed and stained *ex vivo* with fluorescent antibodies for CD127, CCR7 and CD45RA and then stimulated (5hrs) with HEV peptide poolsand HEV‐specific T‐cells were identified by IFNγ production using intracellular cytokine staining. PBMCs taken from patients early after exposure to HEV (1 month: patient 030 pools ACMNL; 046, BMNL; 047, AGMN) or at a late time point after exposure to HEV (16‐22months: patient 104, pools BFMN; 116, CFMN; 034, ABGL) were assessed. (**A**) The percentage of CD4+ or CD8+ HEV‐specific T‐cells expressing CD127 is shown (red dots,early time point post‐exposure to HEV; black dots,late time point post‐exposure to HEV). (**B**) Pie charts show the memory phenotype of CD4+ or CD8+ HEV‐specific T‐cells at an early or late time point after HEV exposure (base median; Tn = naïve‐like, CD45RA+CCR7+; Tem = effector memory, CD45RA‐CCR7‐; Tcm = central memory, CD45RA‐CCR7+; Temra = terminal effector memory, CD45RA+CCR7‐). **C**) Example FACS plots showing the gating strategy to identify CD4+ and CD8+ IFNγ producing HEV‐specific T‐cells and their phenotype, including fluorescence minus one (FMO) stains for CD127 and CD45RA.
