# Supplementary material for: Characterization of the Specificity, Functionality, and Durability of Host T‐Cell Responses Against the Full‐Length Hepatitis E Virus
Source: Hepatology. 2016 Oct 28;64(6):1934–50. doi: 10.1002/hep.28819 (PMC5132006; doi:10.1002/hep.28819)
Supplement: Supplementary file 1 — Supplementary Figure 1: Additional IFNg ELISpot data in HEV Exposed and Unexposed individuals (A) IFN‐ γ ELISpot response to individual peptide pools (A‐I, L‐N), DMSO (negative control) and Concanavlin A (positive control) in immune‐competent, HEV exposed (black dots) and HEV unexposed (open circles) subjects. Each point represents an individuals' response to the corresponding anitgen. Horizontal lines depict median responses. All points represent raw data, without background subtraction. (B) IFN‐ γ ELISpot response to individual peptide pools (A‐I, L‐N) in HEV unexposed subjects. DMSO (negative control) background response has been subtracted for each data point. Horizontal lines represent group medians (+/‐ IQR). (C) Pearson's correlation analysis for total magnitude of IFN‐γ ELISpot response (Y‐axis; log10 scale) with increasing age of individuals at time of sampling (X‐axis; linear scale). Semi‐log regression line has been plotted. (D) Pearson's correlation analysis for number of positive peptide pools (Y‐axis; linear scale) with increasing age of individuals at time of sampling (X‐axis; linear scale). Linear regression line has been plotted. Supplementary Figure 2: The memory phenotype of HEV‐specific T‐cells: A‐C) Frozen PBMCs were thawed and stained ex vivo with fluorescent antibodies for CD127, CCR7 and CD45RA and then stimulated (5hrs) with HEV peptide poolsand HEV‐specific T‐cells were identified by IFNγ production using intracellular cytokine staining. PBMCs taken from patients early after exposure to HEV (1 month: patient 030 pools ACMNL; 046, BMNL; 047, AGMN) or at a late time point after exposure to HEV (16‐22months: patient 104, pools BFMN; 116, CFMN; 034, ABGL) were assessed. (A) The percentage of CD4+ or CD8+ HEV‐specific T‐cells expressing CD127 is shown (red dots,early time point post‐exposure to HEV; black dots,late time point post‐exposure to HEV). (B) Pie charts show the memory phenotype of CD4+ or CD8+ HEV‐specific T‐cells at an early or late [file HEP-64-1934-s001.pdf]

Supplementary Table 1: Baseline demographics of HEV IgG +ve transplant patients with chronic HEV infection.
M=male, F=female; N/A = not applicable; Un= Unknown; cop/ml = copies/ml; MMF = mycophenolate mofetil

Patient Id	Age (years)	Gender	Interval between onset of HEV infection and sampling (mths)	Duration of acute HEV infection (symptom onset to 1st HEV PCR neg) (mths)	HEV RNA (cop/ml)	HEV genotype	Type of organ transplant	Time from transplant to onset of HEV infection (mths)	Immunosuppressive therapy dose/day
51	46	M	7	N/A	6.6×10^5	3f	Renal	69	Tacrolimus, 2 mg<br>MMF 1 g<br>Prednisolone 10 mg
52	67	M	12	N/A	4.8×10^5	3c	Hepatic	93	Tacrolimus 2.5mg
58	51	M	36	N/A	1.9×10^6	3f	Renal	7	Sirolimus 1 mg<br>MMF 1.5 g<br>Prednisone 5 mg
59	32	M	45	N/A	1.8×10^5	3f	Renal	47	Tacrolimus 1 mg<br>MMF 720 mg<br>Prednisone 5 mg
60	54	M	2	N/A	$8.5\text{x}10^6$	3c	Renal	130	Rapamycine 13g<br>MMF 1g
61	55	F	4	N/A	$7.9\text{x}10^5$	3c	Renal	167	Tacrolimus 1.5mg<br>MMF 1g
62	44	M	47	N/A	$3.9\text{x}10^6$	3f	Renal	38	Tacrolimus 2mg<br>MMF 1g<br>Prednisone 10mg
63	34	M	90	N/A	$3.4\text{x}10^5$	3f	Hepatic	15	Ciclosporine 100mg<br>MMF 1g
53	55	M	19	2	Resolved	Un	Renal	20	Tacrolimus 7 mg<br>MMF 1g<br>Prednisone: 5 mg
54	48	M	22	1	Resolved	3f	Hepatic	2	Tacrolimus 1mg<br>MMF 1g<br>Prednisone 5mg
55	30	M	22	4	Resolved	3f	Renal	25	Tacrolimus 3.5 mg<br>MMF 1g<br>Prednisone 5 mg
56	36	M	27	1	Resolved	Un	Renal	88	Everolimus 1.5g<br>MMF 1.5g<br>Prednisone 5mg
57	34	M	31	5	Resolved	3f	Renal	56	Sirolimus 7 mg<br>MMF 1.4g<br>Prednisone 5 mg
65	77	M	5	5	Resolved	3f	Renal	1	Prednisone 30mg
66	61	M	14	3	Resolved	3f	Renal	199	Everolimus 1mg<br>MMF 720mg
67	60	F	11	8	Resolved	3f	Renal	40	Tacrolimus 2mg<br>MMF 1g<br>Prednisone 5mg
68	55	M	17	1	Resolved	3c	Renal	95	Ciclosporine 125mg<br>MMF 2g
69	65	F	35	12	Resolved	3f	Renal	224	Tacrolimus 5mg<br>MMF 1g<br>Prednisone 20mg

Supplementary Table 2: Immune-competent HEV patient information

Patient ID	Age at time of immunology sampling	Gender	HEV Genotype (Un=Unknown)	HEV RNA +ve at time of immunology sampling	Interval between onset of HEV infection and sampling (months)
021	38	M	Un	N	5
022	48	F	Un	N	6
023	65	M	Un	N	8
024	69	M	Un	N	3
025	35	F	Un	N	1
028	22	F	Un	N	51
029	36	M	1	Y	0.3
030	67	M	3	N	1
031	67	M	3	N	0.5
032	77	M	3	Y	0.5
033	87	F	Un	N	3
034	41	M	3	N	0.5
035	59	M	3	N	1
036	77	F	3	N	9
037	41	F	3	N	13
038	64	M	3	N	10
039	45	F	Un	N	8
040	63	M	3	N	2
041	43	F	Un	N	12
042	69	M	3	Y	4
043	69	M	Un	N	2
044	55	M	3	N	3
045	49	M	Un	N	9
046	64	M	Un	N	1
047	59	M	Un	N	1
048	67	M	Un	N	1
049	61	M	Un	N	0.5
050	66	M	3	Y	0.5

Supplementary Table 2: Immune-competent HEV patient information

Patient ID	Age at time of immunology sampling	Gender	HEV Genotype (Un=Unknown)	HEV RNA +ve at time of immunology sampling	Interval between onset of HEV infection and sampling (months)
100	70	F	3	N	139
101	73	F	3	N	106
102	61	M	3	N	36
103	65	F	3	N	15
104	75	M	3	N	17
105	72	M	3	N	27
106	71	M	3	N	78
107	77	M	3	N	6
108	78	M	3	N	21
109	70	F	3	N	4
110	92	F	3	N	0.5
111	83	M	3	N	84
113	64	M	3	N	5
114	66	F	3	N	52
115	61	M	3	N	8
116	79	M	3	N	22

Supplementary Table 3: HEV peptide array sequences

Peptide #	Peptide Sequence	Amino Acid Residues		Peptide Pool	Genomic Region
		From	To		
1	MEAHQFIKAPGITTA	1	15	A	ORF1
2	QFIKAPGITTAIEQA	5	19	A	ORF1
3	APGITTAIEQAALAA	9	23	A	ORF1
4	TTAIEQAALAAANSA	13	27	A	ORF1
5	EQAALAAANSALANA	17	31	A	ORF1
6	LAAANSALANAVVVR	21	35	A	ORF1
7	NSALANAVVVRPFLS	25	39	A	ORF1
8	ANAVVVRPFLSRLQT	29	43	A	ORF1
9	VVRPFLSRLQTEILI	33	47	A	ORF1
10	FLSRLQTEILINLMQ	37	51	A	ORF1
11	LQTEILINLMQPRQL	41	55	A	ORF1
12	ILINLMQPRQLVFRP	45	59	A	ORF1
13	LMQPRQLVFRPEVLW	49	63	A	ORF1
14	RQLVFRPEVLWNHPI	53	67	A	ORF1
15	FRPEVLWNHPIQRVI	57	71	A	ORF1
16	VLWNHPIQRVIHNEL	61	75	A	ORF1
17	HPIQRVIHNELEQYC	65	79	A	ORF1
18	RVIHNELEQYCRARA	69	83	A	ORF1
19	NELEQYCRARAGRCL	73	87	A	ORF1
20	QYCRARAGRCLEVGA	77	91	A	ORF1
21	ARAGRCLEVGAHPRS	81	95	A	ORF1
22	RCLEVGAHPRSINDN	85	99	A	ORF1
23	VGAHPRSINDNPNVL	89	103	A	ORF1
24	PRSINDNPNVLHRCF	93	107	A	ORF1
25	NDNPNVLHRCFLRPV	97	111	A	ORF1
26	NVLHRCFLRPVGRDV	101	115	A	ORF1
27	RCFLRPVGRDVQRWY	105	119	A	ORF1
28	RPVGRDVQRWYSAPT	109	123	A	ORF1
29	RDVQRWYSAPTRGPA	113	127	A	ORF1
30	RWYSAPTRGPAANCR	117	131	A	ORF1
31	APTRGPAANCRRSAL	121	135	A	ORF1
32	GPAANCRRSALRGLP	125	139	A	ORF1
33	NCRRSALRGLPPVDR	129	143	A	ORF1
34	SALRGLPPVDRTYCF	133	147	A	ORF1
35	GLPPVDRTYCFDGFS	137	151	A	ORF1
36	VDRTYCFDGFSRCAF	141	155	A	ORF1
37	YCFDGFSRCAFAAET	145	159	A	ORF1
38	GFSRCAFAAETGVAL	149	163	A	ORF1
39	CAFAAETGVALYSLH	153	167	A	ORF1
40	AETGVALYSLHDLWP	157	171	A	ORF1
41	VALYSLHDLWPADVA	161	175	A	ORF1
42	SLHDLWPADVAEAMA	165	179	A	ORF1
43	LWPADVAEAMARHGM	169	183	A	ORF1
44	DVAEAMARHGMTRLY	173	187	A	ORF1
45	AMARHGMTRLYAALH	177	191	A	ORF1
46	HGMTRLYAALHLPPE	181	195	A	ORF1
47	RLYAALHLPPEVLLP	185	199	A	ORF1
48	ALHLPPEVLLPPGTY	189	203	A	ORF1

Supplementary Table 3: HEV peptide array sequences

Peptide #	Peptide Sequence	Amino Acid Residues		Peptide Pool	Genomic Region
		From	To		
49	PPEVLLPPGTYHTTS	193	207	A	ORF1
50	LLPPGTYHTTSYLLI	197	211	A	ORF1
51	GTYHTTSYLLIHDGD	201	215	A	ORF1
52	TTSYLLIHDGDRAVV	205	219	A and B	ORF1
53	LLIHDGDRAVVTYEG	209	223	A and B	ORF1
54	DGDRAVVTYEGDTSA	213	227	A and B	ORF1
55	AVVTYEGDTSAGYNH	217	231	A and B	ORF1
56	YEGDTSAGYNHDVSI	221	235	A and B	ORF1
57	TSAGYNHDVSILRAW	225	239	A and B	ORF1
58	YNHDVSILRAWIRTT	229	243	A and B	ORF1
59	VSILRAWIRTTKIVG	233	247	A and B	ORF1
60	RAWIRTTKIVGDHPL	237	251	A and B	ORF1
61	RTTKIVGDHPLVIER	241	255	B	ORF1
62	IVGDHPLVIERVRAI	245	259	B	ORF1
63	HPLVIERVRAIGCHF	249	263	B	ORF1
64	IERVRAIGCHFVLLL	253	267	B	ORF1
65	RAIGCHFVLLLTAAP	257	271	B	ORF1
66	CHFVLLLTAAPEPSP	261	275	B	ORF1
67	LLLTAAPEPSPMPYV	265	279	B	ORF1
68	AAPEPSPMPYVPYPR	269	283	B	ORF1
69	PSPMPYVPYPRSTEV	273	287	B	ORF1
70	PYVPYPRSTEVYVRS	277	291	B	ORF1
71	YPRSTEVYVRSIFGP	281	295	B	ORF1
72	TEVYVRSIFGPGGSP	285	299	B	ORF1
73	VRSIFGPGGSPSLFP	289	303	B	ORF1
74	FGPGGSPSLFPSACS	293	307	B	ORF1
75	GSPSLFPSACSTKST	297	311	B	ORF1
76	LFPSACSTKSTFHAV	301	315	B	ORF1
77	ACSTKSTFHAVPVHI	305	319	B	ORF1
78	KSTFHAVPVHIWDRL	309	323	B	ORF1
79	HAVPVHIWDRLMLFG	313	327	B	ORF1
80	VHIWDRLMLFGATLD	317	331	B	ORF1
81	DRLMLFGATLDDQAF	321	335	B	ORF1
82	LFGATLDDQAFCCSR	325	339	B	ORF1
83	TLDDQAFCCSRLMTY	329	343	B	ORF1
84	QAFCCSRLMTYLRGI	333	347	B	ORF1
85	CSRLMTYLRGISYKV	337	351	B	ORF1
86	MTYLRGISYKVTVGA	341	355	B	ORF1
87	RGISYKVTVGALVAN	345	359	B	ORF1
88	YKVTVGALVANEGWN	349	363	B	ORF1
89	VGALVANEGWNASED	353	367	B	ORF1
90	VANEGWNASEDALTA	357	371	B	ORF1
91	GWNASEDALTAVITA	361	375	B	ORF1
92	SEDALTAVITAAYLT	365	379	B	ORF1
93	LTAVITAAYLTICHQ	369	383	B	ORF1
94	ITAAYLTICHQRYLR	373	387	B	ORF1
95	YLTICHQRYLRTQAI	377	391	B	ORF1
96	CHQRYLRTQAISKGM	381	395	B	ORF1

Supplementary Table 3: HEV peptide array sequences

Peptide #	Peptide Sequence	Amino Acid Residues		Peptide Pool	Genomic Region
		From	To		
97	YLRTQAISKGMRRLE	385	399	B	ORF1
98	QAISKGMRRLEVEHA	389	403	B	ORF1
99	KGMRRLEVEHAQKFI	393	407	B	ORF1
100	RLEVEHAQKFITRLY	397	411	B	ORF1
101	EHAQKFITRLYSWLF	401	415	B	ORF1
102	KFITRLYSWLFEKSG	405	419	B	ORF1
103	RLYSWLFEKSGRDYI	409	423	B	ORF1
104	WLFEKSGRDYIPGRQ	413	427	B	ORF1
105	KSGRDYIPGRQLQFY	417	431	B	ORF1
106	DYIPGRQLQFYAQCR	421	435	B	ORF1
107	GRQLQFYAQCRRWLS	425	439	B and C	ORF1
108	QFYAQCRRWLSAGFH	429	443	B and C	ORF1
109	QCRRWLSAGFHLDPR	433	447	B and C	ORF1
110	WLSAGFHLDPRVLVF	437	451	B and C	ORF1
111	GFHLDPRVLVFDESV	441	455	C	ORF1
112	DPRVLVFDESVPCRC	445	459	C	ORF1
113	LVFDESVPCRCRTFL	449	463	C	ORF1
114	ESVPCRCRTFLKKVA	453	467	C	ORF1
115	CRCRTFLKKVAGKFC	457	471	C	ORF1
116	TFLKKVAGKFCCFMR	461	475	C	ORF1
117	KVAGKFCCFMRWLGQ	465	479	C	ORF1
118	KFCCFMRWLGQECTC	469	483	C	ORF1
119	FMRWLGQECTCFLEP	473	487	C	ORF1
120	LGQECTCFLEPAEGL	477	491	C	ORF1
121	CTCFLEPAEGLVGDH	481	495	C	ORF1
122	LEPAEGLVGDHGHDN	485	499	C	ORF1
123	EGLVGDHGHDNEAYE	489	503	C	ORF1
124	GDHGHDNEAYEGSEV	493	507	C	ORF1
125	HDNEAYEGSEVDQAE	497	511	C	ORF1
126	AYEGSEVDQAEPAHL	501	515	C	ORF1
127	SEVDQAEPAHLDVSG	505	519	C	ORF1
128	QAEPAHLDVSGTYAV	509	523	C	ORF1
129	AHLDVSGTYAVHGHQ	513	527	C	ORF1
130	VSGTYAVHGHQLVAL	517	531	C	ORF1
131	YAVHGHQLVALYRAL	521	535	C	ORF1
132	GHQLVALYRALNVPH	525	539	C	ORF1
133	VALYRALNVPHDIAA	529	543	C	ORF1
134	RALNVPHDIAARASR	533	547	C	ORF1
135	VPHDIAARASRLTAT	537	551	C	ORF1
136	IAARASRLTATVELV	541	555	C	ORF1
137	ASRLTATVELVAGPD	545	559	C	ORF1
138	TATVELVAGPDRLEC	549	563	C	ORF1
139	ELVAGPDRLECRTVL	553	567	C	ORF1
140	GPDRLECRTVLGNKT	557	571	C	ORF1
141	LECRTVLGNKTFRTT	561	575	C	ORF1
142	TVLGNKTFRTTVVDG	565	579	C	ORF1
143	NKTFRTTVVDGAHLE	569	583	C	ORF1
144	RTTVVDGAHLEANGP	573	587	C	ORF1

Supplementary Table 3: HEV peptide array sequences

Peptide #	Peptide Sequence	Amino Acid Residues		Peptide Pool	Genomic Region
		From	To		
145	VDGAHLEANGPEQYV	577	591	C	ORF1
146	HLEANGPEQYVLSFD	581	595	C	ORF1
147	NGPEQYVLSFDASRQ	585	599	C	ORF1
148	QYVLSFDASRQSMGA	589	603	C	ORF1
149	SFDASRQSMGAGSHN	593	607	C	ORF1
150	SRQSMGAGSHNLTYE	597	611	C	ORF1
151	MGAGSHNLTYELTPA	601	615	C	ORF1
152	SHNLTYELTPAGLQV	605	619	C	ORF1
153	TYELTPAGLQVRISS	609	623	D	ORF1
154	TPAGLQVRISSNGLD	613	627	D	ORF1
155	LQVRISSNGLDCTAT	617	631	D	ORF1
156	ISSNGLDCTATFPPG	621	635	D	ORF1
157	GLDCTATFPPGGAPS	625	639	D	ORF1
158	TATFPPGGAPSAAPG	629	643	D	ORF1
159	PPGGAPSAAPGEVAA	633	647	D	ORF1
160	APSAAPGEVAAFCGA	637	651	D	ORF1
161	APGEVAAFCGALYRY	641	655	D	ORF1
162	VAAFCGALYRYNRFT	645	659	D	ORF1
163	CGALYRYNRFTQRHS	649	663	D	ORF1
164	YRYNRFTQRHSLTGG	653	667	D	ORF1
165	RFTQRHSLTGGLWLH	657	671	D	ORF1
166	RHSLTGGLWLHPEGL	661	675	D	ORF1
167	TGGLWLHPEGLLGIF	665	679	D	ORF1
168	WLHPEGLLGIFPPFS	669	683	D	ORF1
169	EGLLGIFPPFSPGHI	673	687	D	ORF1
170	GIFPPFSPGHIWESA	677	691	D	ORF1
171	PFSPGHIWESANPFC	681	695	D	ORF1
172	GHIWESANPFCGEGT	685	699	D	ORF1
173	ESANPFCGEGTLYTR	689	703	D	ORF1
174	PFCGEGTLYTRTWST	693	707	D	ORF1
175	EGTLYTRTWSTSGFS	697	711	D	ORF1
176	YTRTWSTSGFSSDFS	701	715	D	ORF1
177	WSTSGFSSDFSPPEA	705	719	D	ORF1
178	GFSSDFSPPEAAAPA	709	723	D	ORF1
179	DFSPPEAAAPASAAA	713	727	D	ORF1
180	PEAAAPASAAAPGLP	717	731	D	ORF1
181	APASAAAPGLPHPTP	721	735	D	ORF1
182	AAAPGLPHPTPPVSD	725	739	D	ORF1
183	GLPHPTPPVSDIWAL	729	743	D	ORF1
184	PTPPVSDIWALPPPS	733	747	D	ORF1
185	VSDIWALPPPSEESQ	737	751	D	ORF1
186	WALPPPSEESQVDAA	741	755	D	ORF1
187	PPSEESQVDAASVPL	745	759	D	ORF1
188	ESQVDAASVPLTLVP	749	763	D	ORF1
189	DAASVPLTLVPAGSP	753	767	D	ORF1
190	VPLTLVPAGSPNPIV	757	771	D	ORF1
191	LVPAGSPNPIVLPPP	761	775	D	ORF1
192	GSPNPIVLPPPPPP	765	779	D	ORF1

Supplementary Table 3: HEV peptide array sequences

Peptide #	Peptide Sequence	Amino Acid Residues		Peptide Pool	Genomic Region
		From	To		
193	PIVLPPPPPPPPVRK	769	783	D	ORF1
194	PPPPPPPPVRKPSTP	773	787	D	ORF1
195	PPPPVRKPSTPPPSR	777	791	D	ORF1
196	VRKPSTPPPSRTRRL	781	795	D	ORF1
197	STPPPSRTRRLLYTY	785	799	D	ORF1
198	PSRTRRLLYTYPDGA	789	803	D	ORF1
199	RRLLYTYPDGAKVYA	793	807	E	ORF1
200	YTYPDGAKVYAGSLF	797	811	E	ORF1
201	DGAKVYAGSLFESDC	801	815	E	ORF1
202	VYAGSLFESDCDWLV	805	819	E	ORF1
203	SLFESDCDWLVNASN	809	823	E	ORF1
204	SDCDWLVNASNPGHR	813	827	E	ORF1
205	WLVNASNPGHRPGGG	817	831	E	ORF1
206	ASNPGHRPGGGLCHA	821	835	E	ORF1
207	GHRPGGGLCHAFHQR	825	839	E	ORF1
208	GGGLCHAFHQRFPEA	829	843	E	ORF1
209	CHAFHQRFPEAFYWT	833	847	E	ORF1
210	HQRFPEAFYWTEFIM	837	851	E	ORF1
211	PEAFYWTEFIMREGL	841	855	E	ORF1
212	YWTEFIMREGLAAYT	845	859	E	ORF1
213	FIMREGLAAYTLTPR	849	863	E	ORF1
214	EGLAAYTLTPRPIIH	853	867	E	ORF1
215	AYTLTPRPIIHAVAP	857	871	E	ORF1
216	TPRPIIHAVAPDYRV	861	875	E	ORF1
217	IIHAVAPDYRVEQNP	865	879	E	ORF1
218	VAPDYRVEQNPKRLE	869	883	E	ORF1
219	YRVEQNPKRLEAAYR	873	887	E	ORF1
220	QNPKRLEAAYRETCS	877	891	E	ORF1
221	RLEAAYRETCSRRGT	881	895	E	ORF1
222	AYRETCSRRGTAAYP	885	899	E	ORF1
223	TCSRRGTAAYPLLGS	889	903	E	ORF1
224	RGTAAYPLLGSGIYQ	893	907	E	ORF1
225	AYPLLGSGIYQVPVS	897	911	E	ORF1
226	LGSGIYQVPVSLSFD	901	915	E	ORF1
227	IYQVPVSLSFDAWER	905	919	E	ORF1
228	PVSLSFDAWERNHRP	909	923	E	ORF1
229	SFDAWERNHRPGDEL	913	927	E	ORF1
230	WERNHRPGDELYLTE	917	931	E	ORF1
231	HRPGDELYLTEPAAA	921	935	E	ORF1
232	DELYLTEPAAAWFEA	925	939	E	ORF1
233	LTEPAAAWFEANKPA	929	943	E	ORF1
234	AAAWFEANKPAQPAL	933	947	E	ORF1
235	FEANKPAQPALTITE	937	951	E	ORF1
236	KPAQPALTITEDTAR	941	955	E	ORF1
237	PALTITEDTARTANL	945	959	E	ORF1
238	ITEDTARTANLALEI	949	963	E	ORF1
239	TARTANLALEIDAAT	953	967	E	ORF1
240	ANLALEIDAATEVGR	957	971	E	ORF1

Supplementary Table 3: HEV peptide array sequences

Peptide #	Peptide Sequence	Amino Acid Residues		Peptide Pool	Genomic Region
		From	To		
241	LEIDAATEVGRACAG	961	975	E	ORF1
242	AATEVGRACAGCTIS	965	979	F	ORF1
243	VGRACAGCTISPGIV	969	983	F	ORF1
244	CAGCTISPGIVHYQF	973	987	F	ORF1
245	TISPGIVHYQFTAGV	977	991	F	ORF1
246	GIVHYQFTAGVPGSG	981	995	F	ORF1
247	YQFTAGVPGSGKSRS	985	999	F	ORF1
248	AGVPGSGKSRSIQQG	989	1003	F	ORF1
249	GSGKSRSIQQGDVDV	993	1007	F	ORF1
250	SRSIQQGDVDVVVVP	997	1011	F	ORF1
251	QQGDVDVVVVPTREL	1001	1015	F	ORF1
252	VDVVVVPTRELRNSW	1005	1019	F	ORF1
253	VVPTRELRNSWRRRG	1009	1023	F	ORF1
254	RELRNSWRRRGFAAF	1013	1027	F	ORF1
255	NSWRRRGFAAFTPHT	1017	1031	F	ORF1
256	RRGFAAFTPHTAARV	1021	1035	F	ORF1
257	AAFTPHTAARVTNGR	1025	1039	F	ORF1
258	PHTAARVTNGRRVVI	1029	1043	F	ORF1
259	ARVTNGRRVVIDEAP	1033	1047	F	ORF1
260	NGRRVVIDEAPSLPP	1037	1051	F	ORF1
261	VVIDEAPSLPPHLLL	1041	1055	F	ORF1
262	EAPSLPPHLLLLHMQ	1045	1059	F	ORF1
263	LPPHLLLLHMQRASS	1049	1063	F	ORF1
264	LLLLHMQRASSVHLL	1053	1067	F	ORF1
265	HMQRASSVHLLGDPN	1057	1071	F	ORF1
266	ASSVHLLGDPNQIPA	1061	1075	F	ORF1
267	HLLGDPNQIPAIDFE	1065	1079	F	ORF1
268	DPNQIPAIDFEHAGL	1069	1083	F	ORF1
269	IPAIDFEHAGLVPAI	1073	1087	F	ORF1
270	DFEHAGLVPAIRPEL	1077	1091	F	ORF1
271	AGLVPAIRPELAPTS	1081	1095	F	ORF1
272	PAIRPELAPTSWWHV	1085	1099	F	ORF1
273	PELAPTSWWHVTHRC	1089	1103	F	ORF1
274	PTSWWHVTHRCPADV	1093	1107	F	ORF1
275	WHVTHRCPADVCELI	1097	1111	F	ORF1
276	HRCPADVCELIRGAY	1101	1115	F	ORF1
277	ADVCELIRGAYPKIQ	1105	1119	F	ORF1
278	ELIRGAYPKIQTTSR	1109	1123	F	ORF1
279	GAYPKIQTTSRVLRS	1113	1127	F	ORF1
280	KIQTTSRVLRSLFWN	1117	1131	F	ORF1
281	TSRVLRSLFWNEPAI	1121	1135	F	ORF1
282	LRSLFWNEPAIGQKL	1125	1139	F	ORF1
283	FWNEPAIGQKLVFTQ	1129	1143	F	ORF1
284	PAIGQKLVFTQAAKA	1133	1147	F	ORF1
285	QKLVFTQAAKAANPG	1137	1151	F	ORF1
286	FTQAAKAANPGAITV	1141	1155	F	ORF1
287	AKAANPGAITVHEAQ	1145	1159	F	ORF1
288	NPGAITVHEAQGATF	1149	1163	F	ORF1

Supplementary Table 3: HEV peptide array sequences

Peptide #	Peptide Sequence	Amino Acid Residues		Peptide Pool	Genomic Region
		From	To		
289	ITVHEAQGATFTETT	1153	1167	F	ORF1
290	EAQGATFTETTVIAT	1157	1171	F	ORF1
291	ATFTETTVIATADAR	1161	1175	F	ORF1
292	ETTVIATADARGLIQ	1165	1179	F	ORF1
293	IATADARGLIQSSRA	1169	1183	F	ORF1
294	DARGLIQSSRAHAIV	1173	1187	F	ORF1
295	LIQSSRAHAIVALTR	1177	1191	F	ORF1
296	SRAHAIVALTRHTEK	1181	1195	F	ORF1
297	AIVALTRHTEKCVIL	1185	1199	F	ORF1
298	LTRHTEKCVILDAPG	1189	1203	F	ORF1
299	TEKCVILDAPGLLRE	1193	1207	F	ORF1
300	VILDAPGLLREVGIS	1197	1211	F	ORF1
301	APGLLREVGISDVIV	1201	1215	F	ORF1
302	LREVGISDVIVNNFF	1205	1219	F	ORF1
303	GISDVIVNNFFLAGG	1209	1223	F	ORF1
304	VIVNNFFLAGGEVGH	1213	1227	F and G	ORF1
305	NFFLAGGEVGHHRPS	1217	1231	F and G	ORF1
306	AGGEVGHHRPSVIPR	1221	1235	G	ORF1
307	VGHHRPSVIPRGNPD	1225	1239	G	ORF1
308	RPSVIPRGNPDQNLG	1229	1243	G	ORF1
309	IPRGNPDQNLGTLQA	1233	1247	G	ORF1
310	NPDQNLGTLQAFPPS	1237	1251	G	ORF1
311	NLGTLQAFPPSCQIS	1241	1255	G	ORF1
312	LQAFPPSCQISAYHQ	1245	1259	G	ORF1
313	PPSCQISAYHQLAEE	1249	1263	G	ORF1
314	QISAYHQLAEELGHR	1253	1267	G	ORF1
315	YHQLAEELGHRPAPV	1257	1271	G	ORF1
316	AEELGHRPAPVAAVL	1261	1275	G	ORF1
317	GHRPAPVAAVLPPCP	1265	1279	G	ORF1
318	APVAAVLPPCPELEQ	1269	1283	G	ORF1
319	AVLPPCPELEQGLLY	1273	1287	G	ORF1
320	PCPELEQGLLYMPQE	1277	1291	G	ORF1
321	LEQGLLYMPQELTVS	1281	1295	G	ORF1
322	LLYMPQELTVSDSVL	1285	1299	G	ORF1
323	PQELTVSDSVLVFEL	1289	1303	G	ORF1
324	TVSDSVLVFELTDIV	1293	1307	G	ORF1
325	SVLVFELTDIVHCRM	1297	1311	G	ORF1
326	FELTDIVHCRMAAPS	1301	1315	G	ORF1
327	DIVHCRMAAPSQRKA	1305	1319	G	ORF1
328	CRMAAPSQRKAVLST	1309	1323	G	ORF1
329	APSQRKAVLSTLVGR	1313	1327	G	ORF1
330	RKAVLSTLVGRYGRR	1317	1331	G	ORF1
331	LSTLVGRYGRRTKLY	1321	1335	G	ORF1
332	VGRYGRRTKLYEAAH	1325	1339	G	ORF1
333	GRRTKLYEAAHSDVR	1329	1343	G	ORF1
334	KLYEAAHSDVRESLA	1333	1347	G	ORF1
335	AAHSDVRESLARFIP	1337	1351	G	ORF1
336	DVRESLARFIPTIGP	1341	1355	G	ORF1

Supplementary Table 3: HEV peptide array sequences

Peptide #	Peptide Sequence	Amino Acid Residues		Peptide Pool	Genomic Region
		From	To		
337	SLARFIPTIGPVQAT	1345	1359	G	ORF1
338	FIPTIGPVQATTCEL	1349	1363	G	ORF1
339	IGPVQATTCELYELV	1353	1367	G	ORF1
340	QATTCELYELVEAMV	1357	1371	G	ORF1
341	CELYELVEAMVEKGQ	1361	1375	G	ORF1
342	ELVEAMVEKGQDGSA	1365	1379	G	ORF1
343	AMVEKGQDGSAVLEL	1369	1383	G	ORF1
344	KGQDGSAVLELDLCN	1373	1387	G	ORF1
345	GSAVLELDLCNRDVS	1377	1391	G	ORF1
346	LELDLCNRDVSRITF	1381	1395	G	ORF1
347	LCNRDVSRITFFQKD	1385	1399	G	ORF1
348	DVSRITFFQKDCNKF	1389	1403	G	ORF1
349	ITFFQKDCNKFTTGE	1393	1407	G	ORF1
350	QKDCNKFTTGETIAH	1397	1411	G	ORF1
351	NKFTTGETIAHGKVG	1401	1415	G	ORF1
352	TGETIAHGKVGQGIS	1405	1419	G	ORF1
353	IAHGKVGQGISAWSK	1409	1423	G	ORF1
354	KVGQGISAWSKTFCA	1413	1427	G	ORF1
355	GISAWSKTFCALFGP	1417	1431	G	ORF1
356	WSKTFCALFGPWFRA	1421	1435	G	ORF1
357	FCALFGPWFRAIEKE	1425	1439	G	ORF1
358	FGPWFRAIEKEILAL	1429	1443	G	ORF1
359	FRAIEKEILALLPPN	1433	1447	G	ORF1
360	EKEILALLPPNVFYG	1437	1451	G	ORF1
361	LALLPPNVFYGDAYE	1441	1455	G	ORF1
362	PPNVFYGDAYEESVF	1445	1459	G	ORF1
363	FYGDAYEESVFAAAV	1449	1463	G	ORF1
364	AYEESVFAAAVSGAG	1453	1467	G	ORF1
365	SVFAAAVSGAGSCMV	1457	1471	H	ORF1
366	AAVSGAGSCMVFEND	1461	1475	H	ORF1
367	GAGSCMVFENDFSEF	1465	1479	H	ORF1
368	CMVFENDFSEFDSTQ	1469	1483	H	ORF1
369	ENDFSEFDSTQNNFS	1473	1487	H	ORF1
370	SEFDSTQNNFSLGLE	1477	1491	H	ORF1
371	STQNNFSLGLECVVM	1481	1495	H	ORF1
372	NFSLGLECVVMEECG	1485	1499	H	ORF1
373	GLECVVMEECGMPQW	1489	1503	H	ORF1
374	VVMEECGMPQWLIRL	1493	1507	H	ORF1
375	ECGMPQWLIRLYHLV	1497	1511	H	ORF1
376	PQWLIRLYHLVRSAW	1501	1515	H	ORF1
377	IRLYHLVRSAWILQA	1505	1519	H	ORF1
378	HLVRSAWILQAPKES	1509	1523	H	ORF1
379	SAWILQAPKESLKGF	1513	1527	H	ORF1
380	LQAPKESLKGFWKKH	1517	1531	H	ORF1
381	KESLKGFWKKHSGEP	1521	1535	H	ORF1
382	KGFWKKHSGEPGTLL	1525	1539	H	ORF1
383	KKHSGEPGTLLWNTV	1529	1543	H	ORF1
384	GEPGTLLWNTVWNMA	1533	1547	H	ORF1

Supplementary Table 3: HEV peptide array sequences

Peptide #	Peptide Sequence	Amino Acid Residues		Peptide Pool	Genomic Region
		From	To		
385	TLLWNTVWNMAIIAH	1537	1551	H	ORF1
386	NTVWNMAIIAHCYEF	1541	1555	H	ORF1
387	NMAIIAHCYEFRDFR	1545	1559	H	ORF1
388	IAHCYEFRDFRVAAF	1549	1563	H	ORF1
389	YEFRDFRVAAFKGDD	1553	1567	H	ORF1
390	DFRVAAFKGDDSVVL	1557	1571	H	ORF1
391	AAFKGDDSVVLCSDY	1561	1575	H	ORF1
392	GDDSVVLCSDYRQSR	1565	1579	H	ORF1
393	VVLCSDYRQSRNAAA	1569	1583	H	ORF1
394	SDYRQSRNAAALIAG	1573	1587	H	ORF1
395	QSRNAAALIAGCGLK	1577	1591	H	ORF1
396	AAALIAGCGLKLKVD	1581	1595	H	ORF1
397	IAGCGLKLKVDYRPI	1585	1599	H	ORF1
398	GLKLKVDYRPIGLYA	1589	1603	H	ORF1
399	KVDYRPIGLYAGVVV	1593	1607	H	ORF1
400	RPIGLYAGVVVAPGL	1597	1611	H	ORF1
401	LYAGVVVAPGLGTLP	1601	1615	H	ORF1
402	VVVAPGLGTLPDVVR	1605	1619	H	ORF1
403	PGLGTLPDVVRFAGR	1609	1623	H	ORF1
404	TLPDVVRFAGRLSEK	1613	1627	H	ORF1
405	VVRFAGRLSEKNWGP	1617	1631	H	ORF1
406	AGRLSEKNWGPGPER	1621	1635	H	ORF1
407	SEKNWGPGPERAEQL	1625	1639	H	ORF1
408	WGPGPERAEQLRLAV	1629	1643	H	ORF1
409	PERAEQLRLAVCDFL	1633	1647	H	ORF1
410	EQLRLAVCDFLRGLT	1637	1651	H	ORF1
411	LAVCDFLRGLTNVAQ	1641	1655	H	ORF1
412	DFLRGLTNVAQVCVD	1645	1659	H	ORF1
413	GLTNVAQVCVDVVSR	1649	1663	H	ORF1
414	VAQVCVDVVSRVYGV	1653	1667	H	ORF1
415	CVDVVSRVYGVSPGL	1657	1671	H	ORF1
416	VSRVYGVSPGLVHNL	1661	1675	H	ORF1
417	YGVSPGLVHNLIGML	1665	1679	H	ORF1
418	PGLVHNLIGMLQTIA	1669	1683	H	ORF1
419	HNLIGMLQTIADGKA	1673	1687	H	ORF1
420	GMLQTIADGKAHFTE	1677	1691	H	ORF1
421	TIADGKAHFTETIKP	1681	1695	H	ORF1
422	GKAHFTETIKPVLDL	1685	1699	H	ORF1
423	FTETIKPVLDLTNSI	1689	1703	H	ORF1
424	IKPVLDLTNSIIQRV	1693	1707	H	ORF1
425	KPVLDLTNSIIQRVE	1694	1708	H	ORF1
426	MNNMFVASPMGSPCA	1	15	I	ORF2
427	FVASPMGSPCALGLF	5	19	I	ORF2
428	PMGSPCALGLFCCCS	9	23	I	ORF2
429	PCALGLFCCCSSCFC	13	27	I	ORF2
430	GLFCCCSSCFCLCCP	17	31	I	ORF2
431	CCSSCFCLCCPRHRP	21	35	I	ORF2
432	CFCLCCPRHRPASRL	25	39	I	ORF2

Supplementary Table 3: HEV peptide array sequences

Peptide #	Peptide Sequence	Amino Acid Residues		Peptide Pool	Genomic Region
		From	To		
433	CCPRHRPASRLAVVV	29	43	I	ORF2
434	HRPASRLAVVVGGAA	33	47	I	ORF2
435	SRLAVVVGGAAAVPA	37	51	I	ORF2
436	VVVGGAAAVPAVVSG	41	55	I	ORF2
437	GAAAVPAVVSGVTGL	45	59	I	ORF2
438	VPAVVSGVTGLILSP	49	63	I	ORF2
439	VSGVTGLILSPSPSP	53	67	I	ORF2
440	TGLILSPSPSPIFIQ	57	71	I	ORF2
441	LSPSPSPIFIQPTPS	61	75	I	ORF2
442	PSPIFIQPTPSPPMS	65	79	I	ORF2
443	FIQPTPSPPMSFHNP	69	83	I	ORF2
444	TPSPPMSFHNPGLEL	73	87	I	ORF2
445	PMSFHNPGLELALGS	77	91	I	ORF2
446	HNPGLELALGSRPAP	81	95	I	ORF2
447	LELALGSRPAPLAPL	85	99	I	ORF2
448	LGSRPAPLAPLGVTS	89	103	I	ORF2
449	PAPLAPLGVTSPSAP	93	107	I	ORF2
450	APLGVTSPSAPPLPP	97	111	I	ORF2
451	VTSPSAPPLPPAVDL	101	115	I	ORF2
452	SAPPLPPAVDLPQLG	105	119	I	ORF2
453	PLPPAVDLPQLGLRR	108	122	I	ORF2
454	MRPRVVLLLFFVFLP	1	15	L	ORF3
455	VVLLLFFVFLPMLPA	5	19	L	ORF3
456	LFFVFLPMLPAPPAG	9	23	L	ORF3
457	FLPMLPAPPAGQPSG	13	27	L	ORF3
458	LPAPPAGQPSGRRRG	17	31	L	ORF3
459	PAGQPSGRRRGRRSG	21	35	L	ORF3
460	PSGRRRGRRSGGAGG	25	39	L	ORF3
461	RRGRRSGGAGGGFWG	29	43	L	ORF3
462	RSGGAGGGFWGDRVD	33	47	L	ORF3
463	AGGGFWGDRVDSQPF	37	51	L	ORF3
464	FWGDRVDSQPFALPY	41	55	L	ORF3
465	RVDSQPFALPYIHPT	45	59	L	ORF3
466	QPFALPYIHPTNPFA	49	63	L	ORF3
467	LPYIHPTNPFAADVV	53	67	L	ORF3
468	HPTNPFAADVVSQSG	57	71	L	ORF3
469	PFAADVVSQSGAGTR	61	75	L	ORF3
470	DVVSQSGAGTRPRQP	65	79	L	ORF3
471	QSGAGTRPRQPPRPL	69	83	L	ORF3
472	GTRPRQPPRPLGSAW	73	87	L	ORF3
473	RQPPRPLGSAWRDQS	77	91	L	ORF3
474	RPLGSAWRDQSQRPS	81	95	L	ORF3
475	SAWRDQSQRPSAAPR	85	99	L	ORF3
476	DQSQRPSAAPRRRSA	89	103	L	ORF3
477	RPSAAPRRRSAPAGA	93	107	L	ORF3
478	APRRRSAPAGAAPLT	97	111	L	ORF3
479	RSAPAGAAPLTAVSP	101	115	M	ORF3
480	AGAAPLTAVSPAPDT	105	119	M	ORF3

Supplementary Table 3: HEV peptide array sequences

Peptide #	Peptide Sequence	Amino Acid Residues		Peptide Pool	Genomic Region
		From	To		
481	PLTAVSPAPDTAPVP	109	123	M	ORF3
482	VSPAPDTAPVPDVDS	113	127	M	ORF3
483	PDTAPVPDVDSRGAI	117	131	M	ORF3
484	PVPDVDSRGAILRRQ	121	135	M	ORF3
485	VDSRGAILRRQYNLS	125	139	M	ORF3
486	GAILRRQYNLSTSPL	129	143	M	ORF3
487	RRQYNLSTSPLTSSV	133	147	M	ORF3
488	NLSTSPLTSSVASGT	137	151	M	ORF3
489	SPLTSSVASGTNLVL	141	155	M	ORF3
490	SSVASGTNLVLYAAP	145	159	M	ORF3
491	SGTNLVLYAAPLNPL	149	163	M	ORF3
492	LVLYAAPLNPLLPLQ	153	167	M	ORF3
493	AAPLNPLLPLQDGTN	157	171	M	ORF3
494	NPLLPLQDGTNTHIM	161	175	M	ORF3
495	PLQDGTNTHIMATEA	165	179	M	ORF3
496	GTNTHIMATEASNYA	169	183	M	ORF3
497	HIMATEASNYAQYRV	173	187	M	ORF3
498	TEASNYAQYRVVRAT	177	191	M	ORF3
499	NYAQYRVVRATIRYR	181	195	M	ORF3
500	YRVVRATIRYRPLVP	185	199	M	ORF3
501	RATIRYRPLVPNAVG	189	203	M	ORF3
502	RYRPLVPNAVGGYAI	193	207	M	ORF3
503	LVPNAVGGYAISISF	197	211	M	ORF3
504	AVGGYAISISFWPQT	201	215	M	ORF3
505	YAISISFWPQTTTTP	205	219	M	ORF3
506	ISFWPQTTTTPTSVD	209	223	M	ORF3
507	PQTTTTPTSVDMNSI	213	227	M	ORF3
508	TTPTSVDMNSITSTD	217	231	M	ORF3
509	SVDMNSITSTDVRIL	221	235	M	ORF3
510	NSITSTDVRILVQPG	225	239	M	ORF3
511	STDVRILVQPGIASE	229	243	M	ORF3
512	RILVQPGIASELVIP	233	247	M	ORF3
513	QPGIASELVIPSERL	237	251	M	ORF3
514	ASELVIPSERLHYRN	241	255	M	ORF3
515	VIPSERLHYRNQGWR	245	259	M	ORF3
516	ERLHYRNQGWRSVET	249	263	M	ORF3
517	YRNQGWRSVETTGVA	253	267	M	ORF3
518	GWRSVETTGVAEEEA	257	271	M	ORF3
519	VETTGVAEEEATSGL	261	275	M	ORF3
520	GVAEEEATSGLVMLC	265	279	M	ORF3
521	EEATSGLVMLCIHGS	269	283	M	ORF3
522	SGLVMLCIHGSPVNS	273	287	M	ORF3
523	MLCIHGSPVNSYTNT	277	291	M	ORF3
524	HGSPVNSYTNTPYTG	281	295	M	ORF3
525	VNSYTNTPYTGALGL	285	299	M	ORF3
526	TNTPYTGALGLLDFA	289	303	M	ORF3
527	YTGALGLLDFALELE	293	307	M	ORF3
528	LGLLDFALELEFRNL	297	311	M	ORF3

Supplementary Table 3: HEV peptide array sequences

Peptide #	Peptide Sequence	Amino Acid Residues		Peptide Pool	Genomic Region
		From	To		
529	DFALELEFRNLTPGN	301	315	M	ORF3
530	ELEFRNLTPGNTNTR	305	319	M	ORF3
531	RNLTPGNTNTRVSRY	309	323	M	ORF3
532	PGNTNTRVSRYTSTA	313	327	M	ORF3
533	NTRVSRYTSTARHRL	317	331	M	ORF3
534	SRYTSTARHRLRRGA	321	335	M	ORF3
535	STARHRLRRGADGTA	325	339	M	ORF3
536	HRLRRGADGTAELTT	329	343	M	ORF3
537	RGADGTAELTTTAAT	333	347	M	ORF3
538	GTAELTTTAATRFMK	337	351	M	ORF3
539	LTTTAATRFMKDLHF	341	355	M	ORF3
540	AATRFMKDLHFTGTN	345	359	M	ORF3
541	FMKDLHFTGTNGVGE	349	363	M	ORF3
542	LHFTGTNGVGEVGRG	353	367	M	ORF3
543	GTNGVGEVGRGIALT	357	371	N	ORF3
544	VGEVGRGIALTLFNL	361	375	N	ORF3
545	GRGIALTLFNLADTL	365	379	N	ORF3
546	ALTLFNLADTLLGGL	369	383	N	ORF3
547	FNLADTLLGGLPTEL	373	387	N	ORF3
548	DTLLGGLPTELISSA	377	391	N	ORF3
549	GGLPTELISSAGGQL	381	395	N	ORF3
550	TELISSAGGQLFYSR	385	399	N	ORF3
551	SSAGGQLFYSRPVVS	389	403	N	ORF3
552	GQLFYSRPVVSANGE	393	407	N	ORF3
553	YSRPVVSANGEPTVK	397	411	N	ORF3
554	VVSANGEPTVKLYTS	401	415	N	ORF3
555	NGEPTVKLYTSVENA	405	419	N	ORF3
556	TVKLYTSVENAQQDK	409	423	N	ORF3
557	YTSVENAQQDKGITI	413	427	N	ORF3
558	ENAQQDKGITIPHDI	417	431	N	ORF3
559	QDKGITIPHDIDLGD	421	435	N	ORF3
560	ITIPHDIDLGDSRVV	425	439	N	ORF3
561	HDIDLGDSRVVIQDY	429	443	N	ORF3
562	LGDSRVVIQDYDNQH	433	447	N	ORF3
563	RVVIQDYDNQHEQDR	437	451	N	ORF3
564	QDYDNQHEQDRPTPS	441	455	N	ORF3
565	NQHEQDRPTPSPAPS	445	459	N	ORF3
566	QDRPTPSPAPSRPFS	449	463	N	ORF3
567	TPSPAPSRPFSVLRA	453	467	N	ORF3
568	APSRPFSVLRANDVL	457	471	N	ORF3
569	PFSVLRANDVLWLSL	461	475	N	ORF3
570	LRANDVLWLSLTAAE	465	479	N	ORF3
571	DVLWLSLTAAEYDQT	469	483	N	ORF3
572	LSLTAAEYDQTTYGS	473	487	N	ORF3
573	AAEYDQTTYGSSTNP	477	491	N	ORF3
574	DQTTYGSSTNPMYVS	481	495	N	ORF3
575	YGSSTNPMYVSDTVT	485	499	N	ORF3
576	TNPMYVSDTVTFVNV	489	503	N	ORF3

Supplementary Table 3: HEV peptide array sequences

Peptide #	Peptide Sequence	Amino Acid Residues		Peptide Pool	Genomic Region
		From	To		
577	YVSDTVTFVNVATGA	493	507	N	ORF3
578	TVTFVNVATGAQAVA	497	511	N	ORF3
579	VNVATGAQAVARSLD	501	515	N	ORF3
580	TGAQAVARSLDWSKV	505	519	N	ORF3
581	AVARSLDWSKVTLDG	509	523	N	ORF3
582	SLDWSKVTLDGRPLT	513	527	N	ORF3
583	SKVTLDGRPLTTIQQ	517	531	N	ORF3
584	LDGRPLTTIQQYSKT	521	535	N	ORF3
585	PLTTIQQYSKTFYVL	525	539	N	ORF3
586	IQQYSKTFYVLPLRG	529	543	N	ORF3
587	SKTFYVLPLRGKLSF	533	547	N	ORF3
588	YVLPLRGKLSFWEAG	537	551	N	ORF3
589	LRGKLSFWEAGTTRA	541	555	N	ORF3
590	LSFWEAGTTRAGYPY	545	559	N	ORF3
591	EAGTTRAGYPYNYNT	549	563	N	ORF3
592	TRAGYPYNYNTTASD	553	567	N	ORF3
593	YPYNYNTTASDQILI	557	571	N	ORF3
594	YNTTASDQILIENAA	561	575	N	ORF3
595	ASDQILIENAAGHRV	565	579	N	ORF3
596	ILIENAAGHRVAIST	569	583	N	ORF3
597	NAAGHRVAISTYTTS	573	587	N	ORF3
598	HRVAISTYTTSLGAG	577	591	N	ORF3
599	ISTYTTSLGAGPTSI	581	595	N	ORF3
600	TTSLGAGPTSISAVG	585	599	N	ORF3
601	GAGPTSISAVGVLAP	589	603	N	ORF3
602	TSISAVGVLAPHSAL	593	607	N	ORF3
603	AVGVLAPHSALAVLE	597	611	N	ORF3
604	LAPHSALAVLEDTVD	601	615	N	ORF3
605	SALAVLEDTVDYPAR	605	619	N	ORF3
606	VLEDTVDYPARAHTF	609	623	N	ORF3
607	TVDYPARAHTFDDFC	613	627	N	ORF3
608	PARAHTFDDFCPECR	617	631	N	ORF3
609	HTFDDFCPECRTLGL	621	635	N	ORF3
610	DFCPECRTLGLQGCA	625	639	N	ORF3
611	ECRTLGLQGCAFQST	629	643	N	ORF3
612	LGLQGCAFQSTIAEL	633	647	N	ORF3
613	GCAFQSTIAELQRLK	637	651	N	ORF3
614	QSTIAELQRLKMKVG	641	655	N	ORF3
615	AELQRLKMKVGKTRE	645	659	N	ORF3
616	ELQRLKMKVGKTRES	649	663	N	ORF3

Supplementary Table 4: In silico epitope prediction using NetMHC software

HEV Peptide No(s)	HEV Peptide Sequence(s)	HEV Peptide Pool	HEV Genomic Region	Patient(s) Responding	Predicted Epitope (SB = Strong Binder, WB = Weak Binder)	Associated HLA restrictions
10	FLSRLQTEILINLMQ	A	ORF1	31	FLSRLQTEI (SB)<br>FLSRLQTEILINLMQ (SB)	A*02<br>DRB1*0101
38/39	GFSRCAFAAETGVAL<br>CAFAAETGVALYSLH	A	ORF1	035, 043	FAAETGVAL (SB) *	C*03
78/79	KSTFHAVPVHIWDRL<br>HAVPVHIWDRLMLFG	B	ORF1	29	VPVHIWDRL (WB) *	B*07
113/114	LVFDESVPCRCRTFL<br>ESVPCRCRTFLKKVA	C	ORF1	030, 031	SVPCRCRTF (SB) *	C*07
199/200	RRLLYTYPDGAKVYA<br>YTYPDGAKVYAGSLF	E	ORF1	113	YPDGAKVYA (SB) *	C*04
260	NGRRVVIDEAPSLPP	F	ORF1	029, 035	NGRRVVIDEAPSLPP (SB)	DRB1*0301
281/282	TSRVLRSLFWNEPAI<br>LRSLFWNEPAIGQKL	F	ORF1	043, 109, 110	SLFWNEPAI (SB) *	A*02
328-330	CRMAAPSQRKAVLST<br>APSQRKAVLSTLVGR<br>RKAVLSTLVGRYGRR	G	ORF1	029, 034, 042, 047, 110	APSQRKAVL (SB)<br>CRMAAPSQRKAVLST (SB)<br>RKAVLSTLVGRYGRR (SB)	B*07<br>DRB5*0101<br>DRB5*0101
432/433	CFCLCCPRHRPASRL<br>CCPRHRPASRLAVVV	I	ORF3	31	CPRHRPASRL (SB) *	B*07
456	LFFVFLPMLPAPPAG	L	ORF2	030, 034	FLPMLPAPPA (SB)<br>LFFVFLPMLPAPPAG (SB)	A*02<br>DPB1*0402
466/467	QPFALPYIHPTNPFA<br>LPYIHPTNPFAADVV	L	ORF2	029, 043, 109, 115	PYIHPTNPF (SB) *<br>QPFALPYIHPTNPFA (WB)	C*03, C*07<br>DPB1*0401
486-488	GAILRRQYNLSTSPL<br>RRQYNLSTSPLTSSV<br>NLSTSPLTSSVASGT	M	ORF2	029, 030, 032, 035, 045, 109, 110, 115	GAILRRQYNLSTSPL (SB)<br>RRQYNLSTSPLTSSV (WB)<br>NLSTSPLTSSVASGT (WB)	DRB4*0101<br>DRB4*0101, DQB1*0602<br>DQB1*0602
491/492	SGTNLVLYAAPLNPL<br>LVLYAAPLNPLLPLQ	M	ORF2	031, 032, 035	YAAPLNPLL (SB)	C*03, C*07
514	ASELVIPSERLHYRN	M	ORF2	035, 108, 110	ASELVIPSERLHYRN (SB)	DRB1*0301
525/526	VNSYTNTPYTGALGL<br>TNTPYTGALGLLDFA	M	ORF2	030, 031, 045, 047, 101, 113	TPYTGALGL (SB) *	B*07, B*35 (WB)
550/551	TELISSAGGQLFYSR<br>SSAGGQLFYSRPVVS	N	ORF2	035, 108	SSAGGQLFY (SB)	A*01
555/556	NGEPTVKLYTSVENA<br>TVKLYTSVENAQQDK	N	ORF2	29	NGEPTVKLYTSVENA (WB)<br>TVKLYTSVENAQQDK (WB)	DRB1*1501<br>DRB5*0101
567	TPSPAPSRPFSVLRA	N	ORF2	031, 045	APSRPFSVL (SB)	B*07
576/577	TNPMYVSDTVTFVNV<br>YVSDTVTFVNVATGA	N	ORF2	31	YVSDTVTFV (SB) *	A*02
587/588	SKTFYVLPLRGKLSF<br>YVLPLRGKLSFWEAG	N	ORF2	31	SKTFYVLPLRGKLSF (SB)<br>YVLPLRGKLSFWEAG (SB)	DRB1*0101<br>DRB1*0101
603/604	AVGVLAPHSALAVLE<br>LAPHSALAVLEDTVD	N	ORF2	31	LAPHSALAVL (SB)	B*07

* In predicted epitope column denotes epitopes that have been successfully tested experimentally

Regions of overlap between consecutive peptides have been underlined.

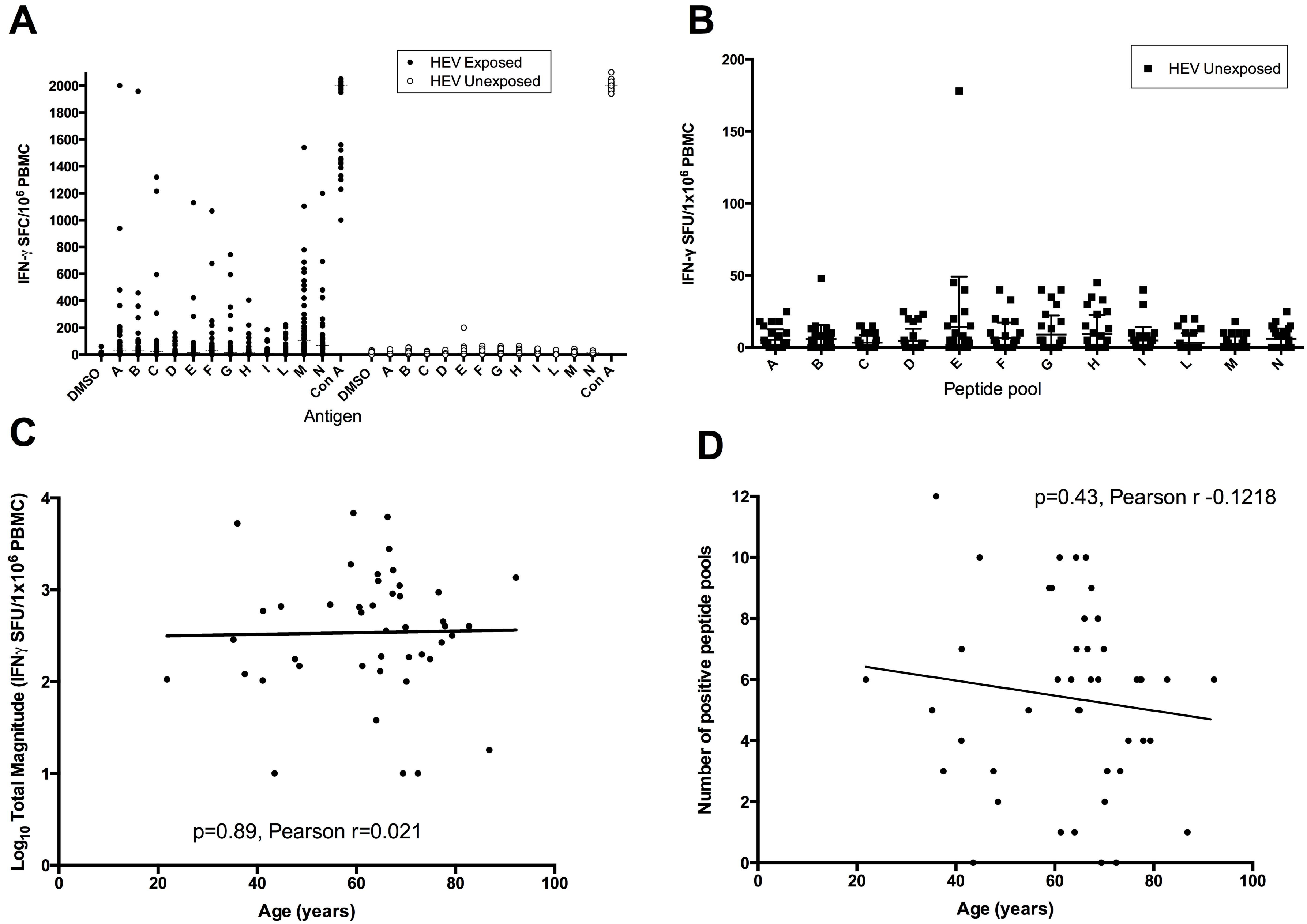
A
HEV Exposed
HEV Unexposed
IFN-γ SFC/10^6 PBMC
Antigen
DMSO A B C D E F G H I L M N Con A DMSO A B C D E F G H I L M N Con A
B
HEV Unexposed
IFN-γ SFU/1x10^6 PBMC
Peptide pool
A B C D E F G H I L M N
C
Log10 Total Magnitude (IFNγ SFU/1x10^6 PBMC)
p=0.89, Pearson r=0.021
Age (years)
D
p=0.43, Pearson r -0.1218
Number of positive peptide pools
Age (years)

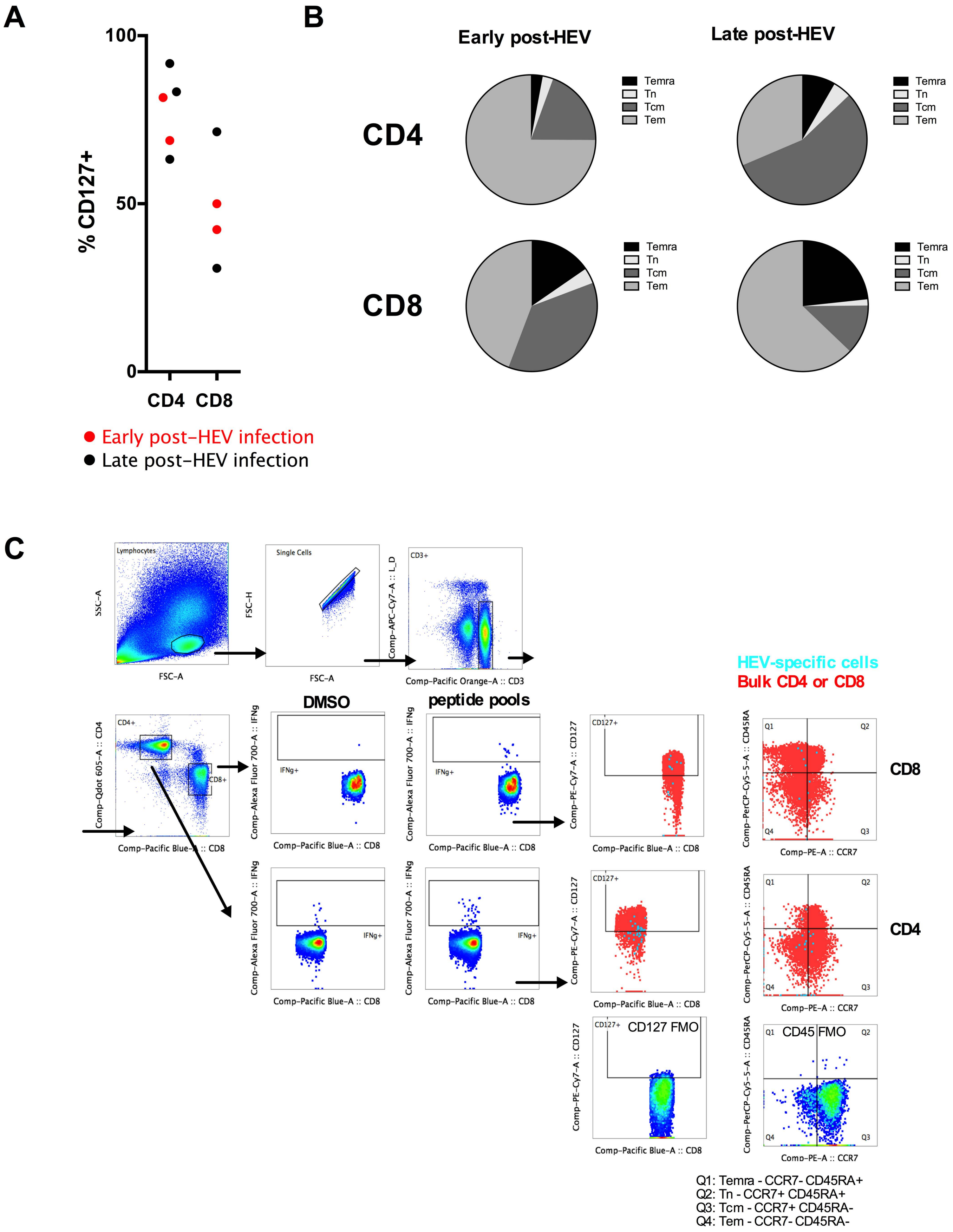
A
100
50
0
% CD127+
CD4
CD8
Early post-HEV infection
Late post-HEV infection
B
Early post-HEV
Late post-HEV
CD4
CD8
Temra
Tn
Tcm
Tem
C
Lymphocytes
SSC-A
FSC-A
Single Cells
FSC-H
CD3+
Comp-APC-Cy7-A :: L_D
Comp-Pacific Orange-A :: CD3
HEV-specific cells
Bulk CD4 or CD8
CD4+
CD8+
Comp-Qdot 605-A :: CD4
Comp-Pacific Blue-A :: CD8
DMSO
peptide pools
IFNg+
Comp-Alexa Fluor 700-A :: IFNg
CD127+
Comp-PE-Cy7-A :: CD127
Q1
Q2
Q3
Q4
Comp-PerCP-Cy5-5-A :: CD45RA
Comp-PE-A :: CCR7
CD127 FMO
CD45 FMO
Q1: Temra - CCR7- CD45RA+
Q2: Tn - CCR7+ CD45RA+
Q3: Tcm - CCR7+ CD45RA-
Q4: Tem - CCR7- CD45RA-
